# Evidence for Centromere Drive in the Holocentric Chromosomes of *Caenorhabditis*

**DOI:** 10.1371/journal.pone.0030496

**Published:** 2012-01-23

**Authors:** František Zedek, Petr Bureš

**Affiliations:** Department of Botany and Zoology, Masaryk University, Brno, Czech Republic; The University of Nottingham, United Kingdom

## Abstract

In monocentric organisms with asymmetric meiosis, the kinetochore proteins, such as CENH3 and CENP-C, evolve adaptively to counterbalance the deleterious effects of centromere drive, which is caused by the expansion of centromeric satellite repeats. The selection regimes that act on CENH3 and CENP-C genes have not been analyzed in organisms with holocentric chromosomes, although holocentrism is speculated to have evolved to suppress centromere drive. We tested both CENH3 and CENP-C for positive selection in several species of the holocentric genus *Caenorhabditis* using the maximum likelihood approach and sliding-window analysis. Although CENP-C did not show any signs of positive selection, positive selection has been detected in the case of CENH3. These results support the hypothesis that centromere drive occurs in Nematoda, at least in the telokinetic meiosis of *Caenorhabditis*.

## Introduction

The centromere ensures proper chromosomal segregation and transmission because it serves as the site of the kinetochore assembly, which mediates the attachment of spindle microtubules. Based on the function and localization of the centromere, eukaryotic chromosomes can be classified into two distinct types: monocentric and holocentric. In mitosis monocentric chromosomes form the kinetochore at a clearly defined region of single primary constriction. In constrast, holocentric chromosomes have spindle microtubules that are attached to the kinetochore, covering most of the poleward surface. During meiosis, the spindle attachment of monocentric chromosomes does not differ from the mitotic condition, whereas microtubules can attach to the kinetochore either at their ends (in organisms with telokinetic meiosis) or along their entire poleward surface (in organisms with holokinetic meiosis) in holocentric chromosomes. Although holocentric chromosomes have evolved independently several times in eukaryotes [1], the mechanism underlying their origin and their potential adaptive value remain unknown.

In general, the centromeres of multicellular eukaryots are composed of large arrays of satellite repeats that are several megabases long [2], and the rapid evolution of centromere satellites is evident from the remarkable variability in the size of arrays between related species [3]. Conversely, the proteins of the kinetochore complex are conserved across all eukaryotes [4]. The centromere-specific variant of histone H3 (CENH3) plays a key role in this complex because it initiates kinetochore formation and establishes the inner kinetochore [5]. Another important protein is CENP-C, which serves as the bridge between the inner and outer kinetochores [5]. Thus, CENH3 and CENP-C are expected to evolve under the strong pressure of negative (purifying) selection. Surprisingly, although most of the CENH3 or CENP-C gene is subjected to negative selection, their DNA-binding domains of either CENH3 or CENP-C have been reported to evolve under positive selection [6].

It has been argued that CENH3 and CENP-C have adaptively evolved to counterbalance the harmful effects of centromere drive [6]. The necessary conditions for this type of drive are (i) asymmetric meiosis (usually female meiosis, where one of four meiotic products survives) and (ii) a difference in the number of emanating microtubules between the egg and the polar body poles [7]. Under the model of centromere drive, the expansion of centromeric satellites leads to the recruitment of more kinetochore proteins and, subsequently, to a “stronger” centromere. A stronger centromere recruits more microtubules, which may confer an advantage, and may be preferentially transmitted to the next generation [8]. However, the spread of the stronger centromere through a population might be accompanied by a number of negative effects, such as aneuploidy, increased male sterility or a skewed sex ratio [8]–[10]. The adaptive mutations of CENH3 or CENP-C weaken the stronger centromeres, thus ensuring balance and suppressing centromere drive [11]. Indeed, the CENH3 and CENP-C have evolved in an adaptive manner in investigated eukaryotic organisms that have displayed asymmetric meiosis [6], [11]. In contrast, the CENH3 and CENP-C in organisms with symmetric meiosis, such as fungi, are evolving under purifying selection [12].

Previous studies have not evaluated the effects of selection on kinetochore proteins in holocentric organisms, even though it has been speculated that such chromosomal status evolves as a defense against centromeric drive [7].

If holocentrism has evolved to suppress centromere drive, holocentric organisms with asymmetric meiosis would resemble organisms with symmetric meiosis, i.e., holocentric organisms would exhibit nonadaptive evolution of kinetochore proteins, at least at some stages of their evolution, depending on the efficiency with which holocentrism can prevent centromere drive. Here, we tested CENH3 and CENP-C for positive selection in *Caenorhabditis*, a genus of Nematoda that possesses holocentric chromosomes with telokinetic meiosis.

## Materials and Methods

The CENH3^HCP-3^ and CENP-C^HCP-4^ sequences of five (*C. japonica*, *C. elegans*, *C. brenneri*, *C. remanei* and *C.briggsae*) *Caenorhabditis* species were obtained from WormBase release WS227 [13]. The sequence of CENH3^HCP-3^ from *Caenorhabditis* species 9 was obtained using BLAST searches of the genomic sequences of the respective species. The WormBase gene IDs and GenBank accession numbers as well as the list of corresponding species are available in Table S1.

The sequences were aligned at the codon level using PRANK [14], [15], as implemented at the Guidance web server [16]. The codon alignments were then examined using Guidance [17], and only those columns with both no gaps and a reliability higher than the default cut-off of 0.93 were used for further analyses. The nucleotide alignments of both CENH3^HCP-3^ and CENP-C^HCP-4^ are available in Text S1 and Text S2, respectively. Unrooted neighbor-joining trees that were based on both the nucleotide alignments and amino acid alignments were constructed in MEGA5 using the Tamura 3-parameter distance and Poisson correction method, respectively, and 1000 bootstrap replicates [18].

We attempted to evaluate positive selection acting on both CENH3^HCP-3^ and CENP-C^HCP-4^ by calculating the nonsynonymous/synonymous substitution rate ratio (dN/dS = ω). Generally, ω<1 indicates purifying selection, ω = 1 suggests neutral evolution, and ω>1 indicates positive selection. First, we tested for positive selection using sliding-window analysis in pairwise sequence comparisons to detect variation in selective pressures along the sequences of both CENH3^HCP-3^ and CENP-C^HCP-4^. This approach was used because positive selection usually acts in short episodes and only on certain subregions of genes. For this reason, the averaging of the ω values for an entire gene to detect positive selection can be misleading. The sliding-window analysis was performed in the K-estimator version 6.1 V [19] with a window size of 15 codons and a step size of 5 codons. For candidate windows with ω>1 a significance of positive selection was then tested using a Z-test as implemented in MEGA5 with default parameters and 1000 bootstrap replicates [18].

We conducted tests of positive selection among the amino acid residues in CENH3^HCP-3^ and CENP-C^HCP-4^ using site models as implemented in PAML4.4 [20]. PAML measures the selective pressure using a maximum-likelihood approach to determine the nonsynonymous/synonymous substitution rate ratio (dN/dS = ω). The sequence data were fitted to pairs of nested models that allow ω to vary among the sites. The simplest model, M0 (one ratio), assumes a uniform ω for all of the sites. M1a (nearly neutral) allows 0<ω<1 for conserved sites and ω = 1 for sites under neutral selection. M2a (positive selection) adds a third class to M1a by allowing ω to be greater than one. Model M3 (discrete) classifies codon sites into three discrete classes of ω estimated from the data. M7 (beta) assumes a beta continuous distribution of ω between 0 and 1, whereas M8 (beta&ω) adds an extra class of sites to the M7 model with ω>1. By comparing M0 and M3, one can determine whether there is one ω for all of the sites (M0) or variation in the ω among the sites (M3). Comparisons of M1a with M2a and M7 with M8 are tests of positive selection (hypotheses M2a and M8). The log-likelihoods of the three pairs of models (M0 vs. M3; M1a vs. M2a; M7 vs. M8) can be compared using the likelihood ratio test (LRT) to identify whether there are significant differences between the two models of each pair. Lastly, to identify the sites that were under positive selection, we used a Bayesian Empirical Bayes (BEB) approach, as implemented in PAML4.4 [20].

PAML was also used to determine selection regimes acting on different branches of a phylogenetic tree by comparing two branch models. The one-ratio model assumes a single ω ratio as an average for all of the branches, whereas the free-ratio model allows a different ω for each branch.

## Results

We analyzed homologs of CENH3 (CENH3^HCP-3^) and CENP-C (CENP-C^HCP-4^) from six (*C. japonica*, *C. elegans*, *C. brenneri*, *C. remanei*, *C. briggsae* and *C. species 9*) and five (*C. japonica*, *C. elegans*, *C. brenneri*, *C. remanei* and *C.briggsae*) *Caenorhabditis* species, respectively. PRANK alignments of the sequences from both genes were inspected by Guidance to identify unreliably aligned regions. The removal of both unreliably aligned regions, as determined by Guidance, and all of the regions containing gaps left 172 codons of CENH3^HCP-3^ and 348 codons of CENP-C^HCP-4^ for further analyses. The alignments of CENH3^HCP-3^ and CENP-C^HCP-4^ both prior and after the removal of unreliable regions are supplied in Texts S1 and S2. The topologies of the inferred phylogenetic trees of CENH3^HCP-3^ (Figure S1) were in agreement with the previously published phylogeny of the genus *Caenorhabditis* [21]. For CENP-C^HCP-4^, only the tree based on nucleotide alignment agreed with the phylogeny of Caenorhabditis species. The tree based on amino acid alignment differed in the switched position of *C. elegans* and *C. brenneri* (Figure S1). However, using of both trees in further analyses gave the same results. Thus, from now on we report the results from the tree of CENP-C^HCP-4^, which was based on nucleotide alignments.

We then applied sliding-window analysis to the alignments of both genes. For CENH3^HCP-3^ only, two adjacent windows (codons 101–115 and 106–120) from the comparison of *C. briggsae* and *C. species 9* exhibited significant signs of positive selection (Z-test, p = 0.011 for both windows). This result suggests that this region, which contains loop 1 of the histone fold domain, experienced an episode of positive selection after the divergence of *C. briggsae* and *C. species 9* from their common ancestor.

**Figure 1:**
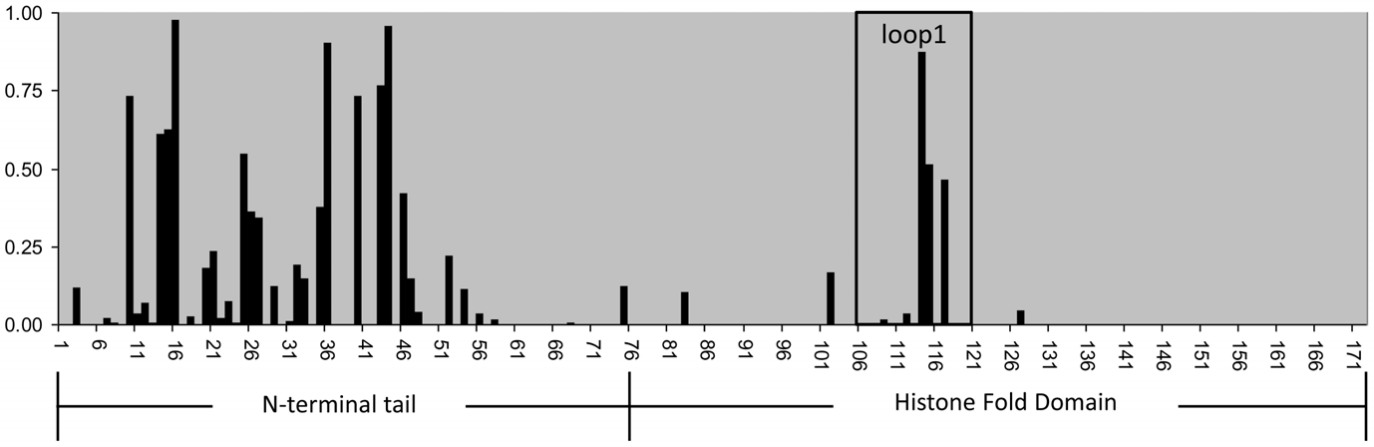
Posterior probabilities that each residue of the CENH3^HCP-3^ is positively selected under M8 model.

To estimate the selection regimes acting on the amino acid residues of CENH3^HCP-3^ and CENP-C^HCP-4^, we used PAML4.4 to compare different site models of codon substitution. The results are shown in Table 1. The estimation of the value of ω as an average across all of the sites and throughout evolutionary history (M0) suggested that both genes, as a whole, are evolving under purifying selection (Table 1). However, a uniform ω ratio for all of the amino acid positions of a gene is rather unexpected in nature. Indeed, the LRT between M0 and M3, the model allowing variation in ω among the sites, showed that M3 furnishes a significantly better fit to the data for both CENH3^HCP-3^ and CENP-C^HCP-4^ (Table 1). This result suggests the possibility that certain sites were adaptively evolving. To test for the occurrence of such sites, we used LRTs to compare the neutral models M1a and M7 with the models allowing positive selection, M2a and M8, respectively. For CENP-C^HCP-4^, the likelihood differences between M1a and M2a or between M7 and M8 were not significant, and, thus, the null hypothesis concerning the neutral evolution of CENP-C^HCP-4^ could not be rejected (Table 1). In contrast, the same LRTs for CENH3^HCP-3^ suggested some regions of this gene were adaptively evolving (Table 1, Figure 1). The positively selected sites of CENH3^HCP-3^ identified by model M8 are shown in Table 1. Model M8 identified eleven positively selected sites. Nine of these sites (positions 10, 14, 15, 16, 25, 36, 40, 43 and 44) are in the N-terminal tail. Two sites (positions 114 and 115) lie in the loop 1 region (L1) of the histone fold domain (Figure 1), which is in accordance with sliding-window analysis (see above). These results imply that CENH3^HCP-3^ is adaptively evolving.

**Table 1:** Results of PAML model comparisons.

	likelihood ratio tests of model comparisons			parameters estimates		positively selected sites
	−2Δl	−2Δl	−2Δl	M0	M8	M8
gene	M0/M3	M1a/M2a	M7/M8			
CENH3^HCP-3^	218**	6.64*	15.85**	ω = 0.12	p1 = 0.09, ω = 5.30	10, 14, 15, **16**, 25, **36**, 40, 43, **44**, 114, 115
					p0 = 0.91, ω≤1	
CENP-C^HCP-4^	114**	0	0	ω = 0.10	NA	NA

Likelihood ratio statistics (2Δl) for comparing models of variable ω among sites. Significant differences are shown in boldface. The significance level is indicated by one (p<0.05) or two (p<0.01) asterisks. Sites with posterior probabilities >90% are in bold face, 70–89% are underlined and 50–69% are in regular font.

**Figure 2:**
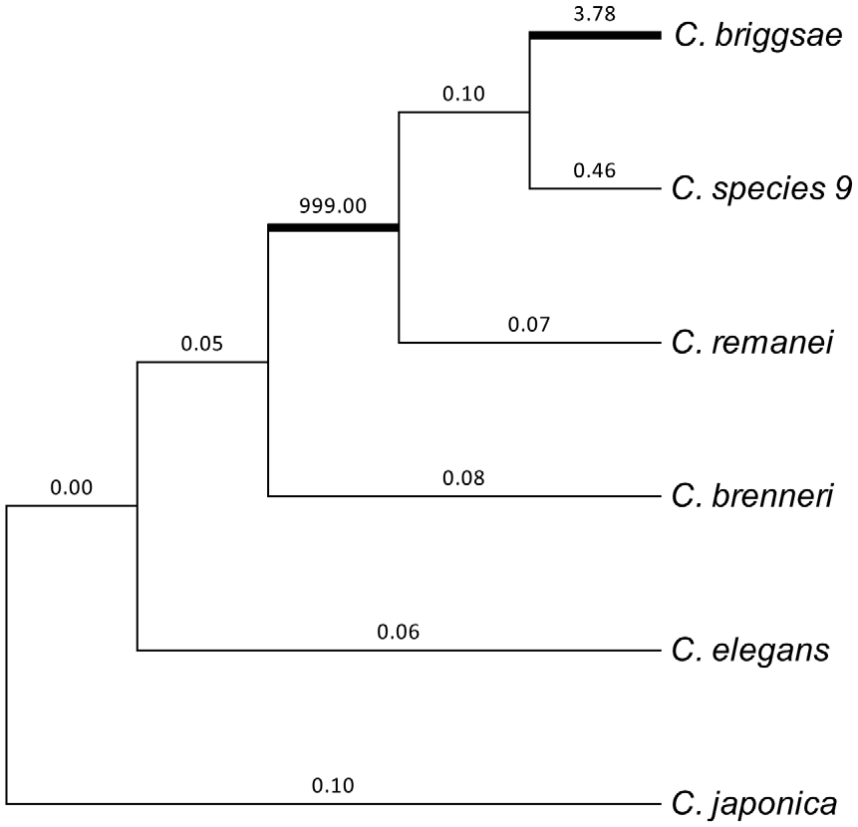
Neighbor-joinning tree of Caenorhabditis CENH3^HCP-3^ gene. Bold branches indicate the lineages under positive selection. Numbers above branches are the ω values. The value of ω = 999 indicates the branch for which dS = 0.

We further tested whether there are differential selective forces among the lineages by comparing two branch models in PAML4.4. The free-ratio model, which allows ω to vary among branches, was significantly better than the one-ratio model only for the CENH3^HCP-3^ gene (−2Δl = 28.84, p = 0.0003). According to the free-ratio model, the branch ancestral to *C. remanei*, *C. briggsae* and *C. species 9* and the branch of *C. briggsae* after the divergence from the common ancestor with *C. species 9* were subjected to positive selection (Figure 2). The remaining lineages were associated with strong purifying selection (Figure 2).

## Discussion

This study is the first attempt to evaluate the selection regimes acting on kinetochore proteins in the holocentric genus, *Caenorhabditis*. Our results indicate that although CENP-C^HCP-4^ has been evolving under negative selection, CENH3^HCP-3^ has undergone episodes of positive selection (Figures 1 and 2). We found positively selected sites in the N-terminal tail of CENH3^HCP-3^ and in a region of the histone fold domain that corresponds well to loop1 of *Drosophila* [22]. Loop1 directly binds to centromeric DNA, and the N-terminal tail has been hypothesized to have a stabilizing function by binding to linker DNA in a similar fashion as canonical histone H3 [22], [23]. Adaptive evolution in these regions suggests recurrent cycles of centromere drive and its suppression [24]. In *Caenorhabditis*, this conflict would be expected to move toward the chromosome ends [7], because they have kinetic activity in the telokinetic meiosis of Nematoda [25], [26]. Indeed, in comparison with the central parts, the chromosomal ends of ascarid nematodes are occupied by abundant repetitive sequences, including satellite repeats [25], [27], which are thought to be involved in centromere function at the chromosomal ends of *C. elegans* [28]. The occurrence of centromere drive at the chromosomal ends in *Caenorhabditis* might be supported by a comparison of the *C. elegans* and *C. briggsae* genomes. These species are morphologically and ecologicaly hardly distinguishible, they have the same number of chromosomes and their genomes exhibit high colinearity [29]. The genome of *C. briggsae* is roughly 4 Mb bigger than the genome of *C. elegans* and this difference is almost entirely due to repetitive sequences, which are non-randomly distributed towards chromosomal ends (see Poster S1 in [29]). Taken together with our observation that CENH3^HCP-3^ has been adaptively evolving in the lineages of both *C. briggsae* and its ancestor (Figure 2), it is possible that the repetitive sequences in the *C. briggsae* genome might have accumulated via centromere drive.

An argument against CENH3^HCP-3^ involvement in the suppression of centromere drive may be the observation of CENH3^HCP-3^-independent meiosis in *C. elegans* [30]. However, this independence does not seem to be universal because meiotic defects were also observed in CENH3^HCP-3^-depleted embryos of *C. elegans* [31]. A recent study by Shakes et al. has suggested that CENH3^HCP-3^ is required in *C. elegans* meiosis, especially during meiosis I [32], which usually determines whether a chromosome will be transferred to the egg. In addition, CENH3^HCP-3^ is present in higher amounts during oogenesis than during spermatogenesis in *C. elegans* [32]. Because oogenesis is an asymmetric meiosis, a necessary condition for centromere drive, the detection of positive selection acting on CENH3^HCP-3^ (Table 1) may support its utilization in *Caenorhabditis* meiosis. However, centromere drive in *Caenorhabditis* might be suppressed regardless of the selection pressure acting on kinetochore proteins or their requirement in meiosis. In the telokinetic meiosis of *C. elegans*, the crossover position determines which end of the chromosome takes over the role of the centromere in meiosis I [33]. Because of this stochasticity, the chances of DNA satellites to affect their fate in asymmetric meiosis are reduced by 50%, implying that the holocentric nature of chromosomes suppresses the centromere drive.

Our results support the hypothesis that the centromere drive occurs in Nematoda [7]. Chromosomal behavior in telokinetic meiosis of *Caenorhabditis* resembles that of monocentric chromosomes, because microtubules are attached to localized regions at chromosome ends and thus expansion of satellite repeats can create a “stronger” centromere. Further studies should focus on holocentric organisms with holokinetic meiosis such as the plant genus *Luzula* (Juncaceae), where the expansion of satellite repeats would seem to have limited function because the kinetochores assemble along the entire chromosome. *Luzula nivea*, for example, has centromere satellite arrays that are approximately 50 kb in size, which is smaller than any other reported centromeric satellite array in plants [34].

## Supporting Information

Figure S1 **The topologies of the inferred phylogenetic trees of both CENH3^HCP-3^ and CENP-C^HCP-4^.**
(PDF)

Table S1 **The WormBase gene IDs and GenBank accession numbers and the list of corresponding species.**
(XLS)

Text S1 **The alignment of CENH3^HCP-3^ both prior and after the removal of unreliable regions.**
(DOC)

Text S2 **The alignment of CENP-C^HCP-4^ both prior and after the removal of unreliable regions.**
(DOC)
