# Correction: Evidence for Centromere Drive in the Holocentric Chromosomes of *Caenorhabditis*

**DOI:** 10.1371/journal.pone.0147889

**Published:** 2016-01-26

**Authors:** František Zedek, Petr Bureš

There are errors in the Supporting Information file S1 Text. Please see the corrected S1 Text here.

## Supporting Information

S1 Text The alignment of CENH3^HCP-3^ both prior and after the removal of unreliable regions. (DOC)
