# Supplementary material for: Evidence for Centromere Drive in the Holocentric Chromosomes of Caenorhabditis
Source: PLoS One. 2012 Jan 23;7(1):e30496. doi: 10.1371/journal.pone.0030496 (PMC3264583; doi:10.1371/journal.pone.0030496)
Supplement: Figure S1 — The topologies of the inferred phylogenetic trees of both CENH3HCP-3 and CENP-CHCP-4. (PDF) [file pone.0030496.s001.pdf]

CENH3^{HCP3} nucleotide based tree

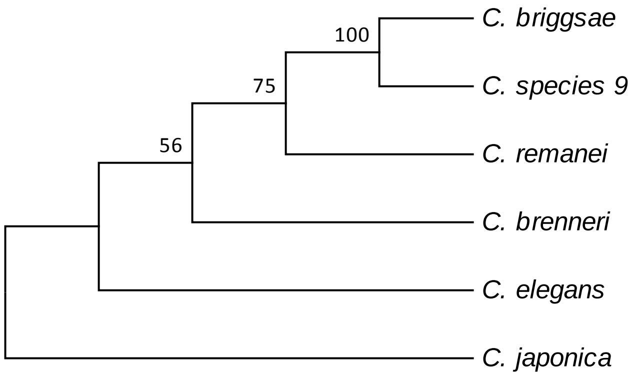

CENH3^{HCP3} amino acid based tree

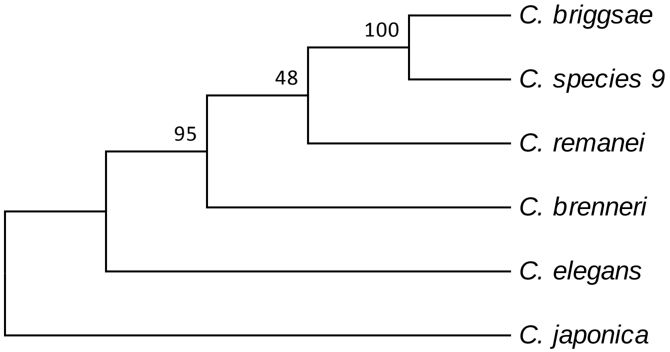

CENP-C^{HCP4} nucleotide based tree

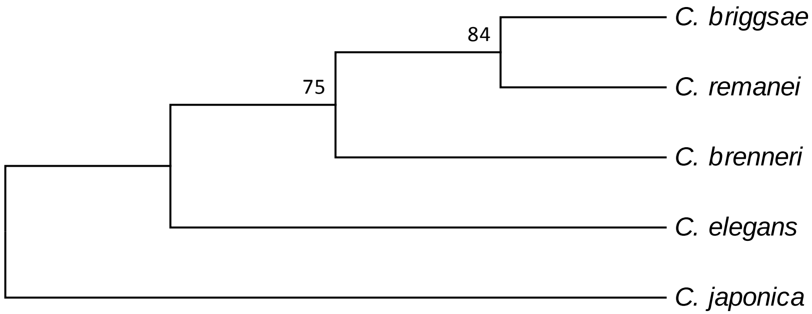

CENP-C^{HCP4} amino acid based tree

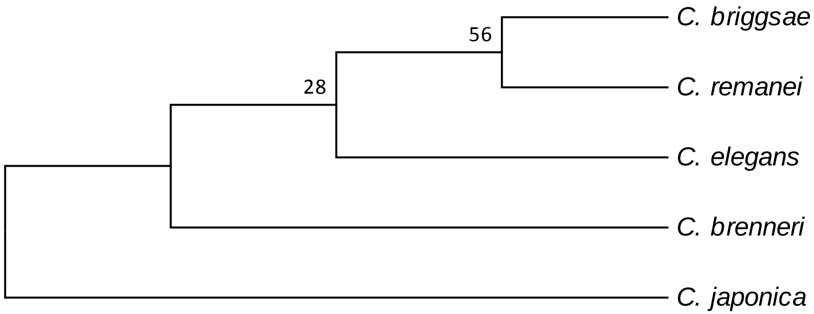

Supplementary File 3: Topologies of CENH3^{HCP3} and CENP-C^{HCP4} sequences inferred from nucleotide and amino acid alignments. The numbers above branches indicate bootstrap values.
