# Supplementary material for: Evidence for Centromere Drive in the Holocentric Chromosomes of Caenorhabditis
Source: PLoS One. 2012 Jan 23;7(1):e30496. doi: 10.1371/journal.pone.0030496 (PMC3264583; doi:10.1371/journal.pone.0030496)
Supplement: Text S1 — The alignment of CENH3HCP-3 both prior and after the removal of unreliable regions. (DOC) [file pone.0030496.s003.doc]

C.elegans ATGGCCGAT------------------GACACCCCAATTATTGAGGAAATCGCCGAGCAA

C.remanei ATGCACCAC------------------AATGGACCGCGTATTGAAGAAATGGTGGATCCA

C.species9 ATGTATCATCATGAC------------AGCGGCCCGCACATTGAAGAAGTTTTTGATCCA

C.briggsae ATGTACCATCATGAC------------AGCGGCCCGCACATTGAAGAAGTTTTTGATCCA

C.brenneri ATGTTTCATCTCTCG------------GATGGTCCCACCATCGAAGAATTAGTGGACACA

C.japonica ATGCAACGA------ATGATAGAGATGGGCGGGCCTCACATAGAGGAAATTGTCGATCCG

PS_C.elegans ------------------------------ACCCCAATTATTGAGGAAATCGCCGAG---

PS_C.remanei ------------------------------GGACCGCGTATTGAAGAAATGGTGGAT---

PS_C.species9 ------------------------------GGCCCGCACATTGAAGAAGTTTTTGAT---

PS_C.briggsae ------------------------------GGCCCGCACATTGAAGAAGTTTTTGAT---

PS_C.brenneri ------------------------------GGTCCCACCATCGAAGAATTAGTGGAC---

PS_C.japonica ------------------------------GGGCCTCACATAGAGGAAATTGTCGAT---

C.elegans AATGAGAGCGTCACAAGGATCATGCAACGTCTC------------------------AAA

C.remanei CCG---TCCAGGAGCACAACAAACCAG---TTA------------------------AAA

C.species9 CCACCGTCTCAAGAAACGATGCTTCGGGAAATA------------------------GCA

C.briggsae CCG---TCTCGACGAACGATGATGCAGGAAATA------------------------GAA

C.brenneri CAACAATTAGAAAACACAGCAGAAGCTGAATTC------------------------AAA

C.japonica CCGAGCCCGAGCAATTCTGTGCTTCAAGAAGCCGATTATCGACAAAATGGTCCGTCTCGC

PS_C.elegans ------------------------------------------------------------

PS_C.remanei ------------------------------------------------------------

PS_C.species9 ------------------------------------------------------------

PS_C.briggsae ------------------------------------------------------------

PS_C.brenneri ------------------------------------------------------------

PS_C.japonica ------------------------------------------------------------

C.elegans CATGAC---------------------------ATGCAAAGAGTCACTTCAGTGCCGGGA

C.remanei AATGATACAGAGTATATCAAA------TCAGAATATCGCAGAATCAGCCATCTTCCAGAC

C.species9 TCCCATCCCGATGTAATTGCACTCTCCAAAAAAGTCCGAAAAATCACAAAAATGCCGGAC

C.briggsae ACTCATCCCGATGTAATTGCATTCGGCAAAAAACTCCGAAAAATTAAAAACCAACCGGAG

C.brenneri GAAGAGCTTGATGTCATTAAG------AAAGAACTGGCAGCTGTCCTTGCCATTCCGGAT

C.japonica AGTCGGCCAAAACTTATC---------GATCAAATTAGAACTCTTATTCGCACGCCAAAT

PS_C.elegans ---------------------------------ATGCAAAGAGTCACTTCAGTGCCGGGA

PS_C.remanei ---------------------------------TATCGCAGAATCAGCCATCTTCCAGAC

PS_C.species9 ---------------------------------GTCCGAAAAATCACAAAAATGCCGGAC

PS_C.briggsae ---------------------------------CTCCGAAAAATTAAAAACCAACCGGAG

PS_C.brenneri ---------------------------------CTGGCAGCTGTCCTTGCCATTCCGGAT

PS_C.japonica ---------------------------------ATTAGAACTCTTATTCGCACGCCAAAT

C.elegans ------TTCAACACAAGCGCCGCAGGTGTCAACGATTTGATCGACATTCTG---AACCAG

C.remanei ------TTCAACAGAGACCCAGAATTAATCCAGGAGGTTATGAATTTGACAAAAAGATAC

C.species9 TCTGCCTTCATTTCTAGTGCCGATCGCCTAGTAGAAATAATCGATGCGTTTAGCGAGCAA

C.briggsae TCCACCTTTCTTTCTAGTGCCGATCGCATGGAAGAAATAATCGATGCGTTTAGAGATCAA

C.brenneri ------ATCCACAGAAACCGTGAAGCATTGGAAAAAAGTATCCGCATTCTAGAGAAAGCT

C.japonica ------TTCAACAAAGACGCCGTCAAGATGGGTCAAGCTATCGATCTAATGGAGCTGCAA

PS_C.elegans ------TTCAACACAAGCGCCGCAGGTGTCAACGATTTGATCGACATTCTG---------

PS_C.remanei ------TTCAACAGAGACCCAGAATTAATCCAGGAGGTTATGAATTTGACA---------

PS_C.species9 ------TTCATTTCTAGTGCCGATCGCCTAGTAGAAATAATCGATGCGTTT---------

PS_C.briggsae ------TTTCTTTCTAGTGCCGATCGCATGGAAGAAATAATCGATGCGTTT---------

PS_C.brenneri ------ATCCACAGAAACCGTGAAGCATTGGAAAAAAGTATCCGCATTCTA---------

PS_C.japonica ------TTCAACAAAGACGCCGTCAAGATGGGTCAAGCTATCGATCTAATG---------

C.elegans TATAAGAAGGAGCTTGAGGATGATGCAGCCAACGACTACACTGAAGCGCACATCCACAAA

C.remanei ATCGAAAAGTGGCTACGA---GAAGAAAGAGACGAACCGAATATGGAGCGTCAAGGATGG

C.species9 ATCGAAAAGTGGAAAGAAGATGAAACATTGGACGACCCATGCCCATATCTGTCATTGAAA

C.briggsae ATCGCCAAGTGGGAAGAAGAGGAAGAGTTGAATGAGCCATGTGAATATCGGCAATTAAAA

C.brenneri ATCGATAAATGGGAAGAGGATGAAGAAAACCAAGTATCACTCGAGCTTCGCAGGCAGTCT

C.japonica ATTGCCGAATGGGTTGAGGAGCAAATTCGATACGGATTCACACAGGAACGCGAAGATGCC

PS_C.elegans ------------------------------------------------------------

PS_C.remanei ------------------------------------------------------------

PS_C.species9 ------------------------------------------------------------

PS_C.briggsae ------------------------------------------------------------

PS_C.brenneri ------------------------------------------------------------

PS_C.japonica ------------------------------------------------------------

C.elegans ATTCGATTGGTCACAGGCAAACGGAATCAA------------------------TATGTC

C.remanei ATTGAGCGCTTCAAAACAAAGCTTCGTGAA------------------------TGGGAA

C.species9 ATCGAATTTTTCACAGAAAAGAGAAACCAA------------------------TACCAA

C.briggsae ATTGAAATTTTCACGCAAAAGAAAATTGAA------------------------TACCAA

C.brenneri ATTGGGAAGTTTAAAGAACAGCGTCGTAGC------------------------TGTAAA

C.japonica ATCTACCAGTACAGACGCAAATTGCGACGTCAAAACGCGAAACCTCGCCCACTTTTTTCA

PS_C.elegans ------------------------------------------------------------

PS_C.remanei ------------------------------------------------------------

PS_C.species9 ------------------------------------------------------------

PS_C.briggsae ------------------------------------------------------------

PS_C.brenneri ------------------------------------------------------------

PS_C.japonica ------------------------------------------------------------

C.elegans TTGAAGTTG------------------------AAGCAAGCCGAAGACGAATATCACGCG

C.remanei ACGAAAAAA------------------------GAAACCGCAGAAGATGAATACTATACA

C.species9 AGAAAAAAT------------------------AGTTCGGCAGTTGATCGATACTATGAT

C.briggsae AGAAAAAAT------------------------AATTTGGCAGTCGACGAATTCTATAAG

C.brenneri CAGAAACTC------------------------CGAGACGCTGAAAATGCGTTTCACGAA

C.japonica CGCAAAAAATTCCAAATGTTCAAAAAAAGCGTCGAAGAAGCAGAAGAACGATACTTTGAA

PS_C.elegans ---------------------------------AAGCAAGCCGAAGACGAATATCACGCG

PS_C.remanei ---------------------------------GAAACCGCAGAAGATGAATACTATACA

PS_C.species9 ---------------------------------AGTTCGGCAGTTGATCGATACTATGAT

PS_C.briggsae ---------------------------------AATTTGGCAGTCGACGAATTCTATAAG

PS_C.brenneri ---------------------------------CGAGACGCTGAAAATGCGTTTCACGAA

PS_C.japonica ---------------------------------GAAGAAGCAGAAGAACGATACTTTGAA

C.elegans CGAAAA---------------------------------GAGCAAGCTCGGAGAAGAGCT

C.remanei CGACGAGAC---------------------GCGTCTTCGAATGAAGAGAAAAATAGAGAA

C.species9 GGCAAAGATTCCCGGGATTACAGCAGCAGAAGGCCGTTGGAAGAGAGCCGAAGACGAGAG

C.briggsae AAGAGAAATCTTAAGAATCACAGCAACAGAAAACCGTTGGAAGAGAGCAGGAGAAGAGAG

C.brenneri AGGAGAGAA---------------------CGGGAATATGAAGAAAGGACAATGAGAGAG

C.japonica GAAAGA---------------------------------GAGGAGAGCAGACGGCGAGAA

PS_C.elegans ------------------------------------------------------------

PS_C.remanei ------------------------------------------------------------

PS_C.species9 ------------------------------------------------------------

PS_C.briggsae ------------------------------------------------------------

PS_C.brenneri ------------------------------------------------------------

PS_C.japonica ------------------------------------------------------------

C.elegans ------------------------TCGTCTATGGATTTCACGGTCGGCAGAAATTCCACG

C.remanei ATCGCACGGCGGAGAGCCACCGATTCTCAGATGAATATCACAGGTCTCCACGATAGCACC

C.species9 GAGCCTCGAGATAGAGGTCATGAAACGAATATTGACATAACGCATCGTGGCGATAGCACC

C.briggsae GAGCCTCGAGATAGAGTCCACGAATCGAATATAGACATAACGCATCGTGGCGATAGCACC

C.brenneri ATTCCTCGGAGGTACTCCAGTTTTCGGGATACAGACATAACGAGGCGTAACAATACGACA

C.japonica GAAGCACGCAGAGCAATGAGCTACAGTCGCGGGGACATTTCGGCACGAGACAATCGTTCA

PS_C.elegans ------------------------------ATGGATTTCACGGTCGGCAGAAATTCCACG

PS_C.remanei ------------------------------ATGAATATCACAGGTCTCCACGATAGCACC

PS_C.species9 ------------------------------ATTGACATAACGCATCGTGGCGATAGCACC

PS_C.briggsae ------------------------------ATAGACATAACGCATCGTGGCGATAGCACC

PS_C.brenneri ------------------------------ACAGACATAACGAGGCGTAACAATACGACA

PS_C.japonica ------------------------------GGGGACATTTCGGCACGAGACAATCGTTCA

C.elegans AATCTTGTC---------GAT------------------TACTCCCACGGCCGTCATCAT

C.remanei AGACTGAAT---------CAA------------------CAATCTTATTCACGC------

C.species9 AGTCTGAATCATTATTCGCAGCGACATTATTCTCAACGACAATCACAAAGTTCT------

C.briggsae AGTCTGAATCATTATTCTCGGCACCATTATTCTCAACGACAATCACAAAGTTCT------

C.brenneri GGACTATACCATCATTCTCAG------------------CAAAGTTCTTCTAAT------

C.japonica AAACTACAT---------CAA------------------TCTCACACGCAAAGA------

PS_C.elegans AATCTT------------------------------------------------------

PS_C.remanei AGACTG------------------------------------------------------

PS_C.species9 AGTCTG------------------------------------------------------

PS_C.briggsae AGTCTG------------------------------------------------------

PS_C.brenneri GGACTA------------------------------------------------------

PS_C.japonica AAACTA------------------------------------------------------

C.elegans ATGCCCTCATACCGT---------CGACACGATAGCTCCGACGAAGAA---AACTAT---

C.remanei ------TCCTATGAAAACCGGAATAGAAGATACAGTTCTGATGAAGACGATGATGAG---

C.species9 ------CGATTCGAA---------AGAGATCGTGAATCCGAAGAGAAA---GATGAG---

C.briggsae ------CGGTTCGAA---------AGAGAGCGTGAATCCGACGAGGAA---GAGGAA---

C.brenneri ------TTTCGGATG---------CAAGAATACAGTTCAGACGAAGAA---ATAGAA---

C.japonica ------AATTACGGC---------GATAGCTTGGATTCGGACGATGAG---AATGAGCGT

PS_C.elegans ------------------------------------------------------------

PS_C.remanei ------------------------------------------------------------

PS_C.species9 ------------------------------------------------------------

PS_C.briggsae ------------------------------------------------------------

PS_C.brenneri ------------------------------------------------------------

PS_C.japonica ------------------------------------------------------------

C.elegans ------------------TCTATGGATGGAACAAATGGCGAT------------------

C.remanei ------------------AATATGGCACCCCAGCGACGTCAA------------------

C.species9 ------------------AATAGGCACCCGAGACAACAATAT------------------

C.briggsae ------------------AATAGTCAGCCGATTCAACGTTAT------------------

C.brenneri ------------------AACATTCCAAGCTCACACCGCGAT------------------

C.japonica GAGAATGGTTATCAGAGTTATAGACCGCCTCCACAGAGGCAACAACGTTTGCGTTCACGT

PS_C.elegans ------------------------------------------------------------

PS_C.remanei ------------------------------------------------------------

PS_C.species9 ------------------------------------------------------------

PS_C.briggsae ------------------------------------------------------------

PS_C.brenneri ------------------------------------------------------------

PS_C.japonica ------------------------------------------------------------

C.elegans ---GGAAATAGAGCTGGC------------------------------------------

C.remanei ---CGCTCTCGGTCTCCT------------------------------------------

C.species9 ---CGTTCTAGATCTCCCCAA---------------------------------------

C.briggsae ---CGTTCTAGATCTCCCAAA---------------------------------------

C.brenneri ---CGTTATAGGTTAGAAAAGTGTTTGATTATCGTTTTTCAAAATCTATATTTCAGTTAC

C.japonica TCTCGATCCCGCTCTCCG------------------------------------------

PS_C.elegans ------------------------------------------------------------

PS_C.remanei ------------------------------------------------------------

PS_C.species9 ------------------------------------------------------------

PS_C.briggsae ------------------------------------------------------------

PS_C.brenneri ------------------------------------------------------------

PS_C.japonica ------------------------------------------------------------

C.elegans CCATCGAACCCC------------------------------------GAT---------

C.remanei CCATCGTTTGCT------------------CACCACCAACGTCGAGATGATACCGGGTCG

C.species9 CACACACACAGCTACAACCAATCCACAATGCAT------CAGCGTGATGATACCAACGTT

C.briggsae CCATCATACAGCTACAACCAATCGACAATGCAACAGTCACAACGTGATGATACCAACGTT

C.brenneri CCGCCAAAAAAAATCAGTCATTCAACAATGCTCCAACAACGCCGTGACATTAGCCCGGTT

C.japonica ATGCGGTCGTCGTACCGTCATGAATCGCCCGAAAACAGCCGTAGAAACGCA---------

PS_C.elegans ------------------------------------------------------------

PS_C.remanei ------------------------------------------------------------

PS_C.species9 ------------------------------------------------------------

PS_C.briggsae ------------------------------------------------------------

PS_C.brenneri ------------------------------------------------------------

PS_C.japonica ------------------------------------------------------------

C.elegans ---------CGTGGTAAT------------------------------------------

C.remanei TACTACAGAAGTCATCACACTCAAAATTCTTCAAATCAAAGAACTCATAACACTGATTTT

C.species9 TACCATAGAAGTCATCAA------------------------------------------

C.briggsae TACCATAGAAGCCATCAA------------------------------------------

C.brenneri GTTTATCGTAGTCAGCAA------------------------------------------

C.japonica ---------TCACATCAA------------------------------------------

PS_C.elegans ------------------------------------------------------------

PS_C.remanei ------------------------------------------------------------

PS_C.species9 ------------------------------------------------------------

PS_C.briggsae ------------------------------------------------------------

PS_C.brenneri ------------------------------------------------------------

PS_C.japonica ------------------------------------------------------------

C.elegans ------------------------------AGAACTGGC---------------CCATCG

C.remanei AGCTCGCATTATAGAGGGCAATACGGACCATCAACGTCGCAAAATGTGGGCATGCCATCA

C.species9 ------------------------------AGCACGTCTCAG------------CCTTCA

C.briggsae ------------------------------AGCACATCTCAA------------CCTCCA

C.brenneri ------------------------------CAGAGTTCAGCA------------GGTTCT

C.japonica ------------------------------CAAACCGCACAA------------------

PS_C.elegans ------------------------------------------------------------

PS_C.remanei ------------------------------------------------------------

PS_C.species9 ------------------------------------------------------------

PS_C.briggsae ------------------------------------------------------------

PS_C.brenneri ------------------------------------------------------------

PS_C.japonica ------------------------------------------------------------

C.elegans AGCTCCGATCGCGTGCGGATGAGAGCCGGAAGGAACAGAGTCACCAAAACG------AGA

C.remanei AATGCTCAGAACGTGAGGATGCGCTCAGGAAAAAGCAGAGTCACAAAGACGCGTAGTCGC

C.species9 ------CAA---GTGAGAATGCGTTCTGGAAAAAGCCGTGTCACGAAGACACACAACCGC

C.briggsae ------CAA---GTGAGAATGCGTTCCGGAAAAAGCCGTGTCACGAAGACACACAACCGC

C.brenneri ------CAACAAGAGCGGATGCGATCCGGAAAAAGTCGAGTAACAAAGACA---ACCCGC

C.japonica ------------GTCAGAATGCGTGCCGGAAAGAACAACGTCACGAAAACA------AAA

PS_C.elegans ------------GTGCGGATGAGAGCCGGAAGGAACAGAGTCACCAAA------------

PS_C.remanei ------------GTGAGGATGCGCTCAGGAAAAAGCAGAGTCACAAAG------------

PS_C.species9 ------------GTGAGAATGCGTTCTGGAAAAAGCCGTGTCACGAAG------------

PS_C.briggsae ------------GTGAGAATGCGTTCCGGAAAAAGCCGTGTCACGAAG------------

PS_C.brenneri ------------GAGCGGATGCGATCCGGAAAAAGTCGAGTAACAAAG------------

PS_C.japonica ------------GTCAGAATGCGTGCCGGAAAGAACAACGTCACGAAA------------

C.elegans CGTTATAGACCGGGCCAGAAGGCATTGGAAGAGATCCGCAAGTACCAAAAAACTGAAGAC

C.remanei AAGTGGCGACCTGGACAGAGAGCGCTTGAGGAAATTCGAAAATACCAAAAGTCCACCGAT

C.species9 AAGTTTCGACCCGGACAGAAAGCCTTAGCTGAAATTCGAAAGTATCAGAAGTCGACAGAT

C.briggsae AAGTTTCGACCTGGACAGAAAGCCTTGGCTGAAATTCGAAAGTATCAGAAGTCGACAGAT

C.brenneri AAGCACAGACCGGGGCAAAAAGCGTTGGCAGAGATAAGGAAATACCAGAAGTCAACTGAT

C.japonica AAATGGCGTCCAGGACAGAAGGCGTTGAGTGAGATTCGAAAATACCAAAATTCCACTGAT

PS_C.elegans CGTTATAGACCGGGCCAGAAGGCATTGGAAGAGATCCGCAAGTACCAAAAAACTGAAGAC

PS_C.remanei AAGTGGCGACCTGGACAGAGAGCGCTTGAGGAAATTCGAAAATACCAAAAGTCCACCGAT

PS_C.species9 AAGTTTCGACCCGGACAGAAAGCCTTAGCTGAAATTCGAAAGTATCAGAAGTCGACAGAT

PS_C.briggsae AAGTTTCGACCTGGACAGAAAGCCTTGGCTGAAATTCGAAAGTATCAGAAGTCGACAGAT

PS_C.brenneri AAGCACAGACCGGGGCAAAAAGCGTTGGCAGAGATAAGGAAATACCAGAAGTCAACTGAT

PS_C.japonica AAATGGCGTCCAGGACAGAAGGCGTTGAGTGAGATTCGAAAATACCAAAATTCCACTGAT

C.elegans CTTCTGATTCAAAAGGCTCCGTTCGCACGCCTCGTCCGCGAAATTATGCAGACTTCCACT

C.remanei ATGCTGATTCAGAAAGCTCCCTTTGCACGTCTTGTCCACGAAATTATGCGCGAAGCAACT

C.species9 ATGCTGATCCAGAAGGCTCCTTTTGCTCGTCTTGTTCATGAAATTGTTCGAGAACAAACC

C.briggsae ATGTTGATCCAGAAGGCTCCTTTTGTTCGTCTTGTTCATGAAATTATTCGAGAACAAACC

C.brenneri CTTTTGATTCAGAAAGCTCCATTTGCACGCCTTGTCCATGAAATTATCCGGGAAGCAACT

C.japonica TTGCTCATTCAAAAAGCCCCCTTCCGTCGATTAGTTCACCAGATTATTCAAGAAGCGACC

PS_C.elegans CTTCTGATTCAAAAGGCTCCGTTCGCACGCCTCGTCCGCGAAATTATGCAGACTTCCACT

PS_C.remanei ATGCTGATTCAGAAAGCTCCCTTTGCACGTCTTGTCCACGAAATTATGCGCGAAGCAACT

PS_C.species9 ATGCTGATCCAGAAGGCTCCTTTTGCTCGTCTTGTTCATGAAATTGTTCGAGAACAAACC

PS_C.briggsae ATGTTGATCCAGAAGGCTCCTTTTGTTCGTCTTGTTCATGAAATTATTCGAGAACAAACC

PS_C.brenneri CTTTTGATTCAGAAAGCTCCATTTGCACGCCTTGTCCATGAAATTATCCGGGAAGCAACT

PS_C.japonica TTGCTCATTCAAAAAGCCCCCTTCCGTCGATTAGTTCACCAGATTATTCAAGAAGCGACC

C.elegans CCATTTGGCGCCGACTGCCGTATTCGTTCTGACGCCATCAGTGCTCTTCAAGAAGCGGCG

C.remanei TCGGAAAGTCAAGATTTTCGGATTCGTGCAGACGCTTTGATGGCTCTTCAAGAAGCGGCA

C.species9 AACCAAAGTAAAGACTATCGTATTCGTGCCGATGCTTTGATGGCTCTACAGGAAGCAGCA

C.briggsae TACAAAAGTCAAGACTATCGTATTCGTGCGGATGCTTTGATGGCTCTACAGGAAGCAGCA

C.brenneri ACAAATAGTGGAGATTATCGCGTTCGTGCAGATGCTCTTCTAGCTCTCCAAGAAGGCGCT

C.japonica GGCTTCGATTCCGGATTCCGCATTCGCGCCGACGCGATGTCTGCCCTACAAGAAGCCGCC

PS_C.elegans CCATTTGGCGCCGACTGCCGTATTCGTTCTGACGCCATCAGTGCTCTTCAAGAAGCGGCG

PS_C.remanei TCGGAAAGTCAAGATTTTCGGATTCGTGCAGACGCTTTGATGGCTCTTCAAGAAGCGGCA

PS_C.species9 AACCAAAGTAAAGACTATCGTATTCGTGCCGATGCTTTGATGGCTCTACAGGAAGCAGCA

PS_C.briggsae TACAAAAGTCAAGACTATCGTATTCGTGCGGATGCTTTGATGGCTCTACAGGAAGCAGCA

PS_C.brenneri ACAAATAGTGGAGATTATCGCGTTCGTGCAGATGCTCTTCTAGCTCTCCAAGAAGGCGCT

PS_C.japonica GGCTTCGATTCCGGATTCCGCATTCGCGCCGACGCGATGTCTGCCCTACAAGAAGCCGCC

C.elegans GAAGCATTTTTGGTCGAAATGTTCGAAGGATCGTCTCTTATATCCACCCATGCGAAACGT

C.remanei GAAGCGTTCATGGTGGAGATGTTCGAGGGATCCGTGTTGATTTGTAATCACGCGAAAAGA

C.species9 GAAGCATTCATGGTTGAAATGTTCGAAGGATCCGTTCTGATTTGCAATCACGCTAAGCGT

C.briggsae GAAGCATTCATGGTTGAAATGTTCGAAGGATCCGTACTGATTTGCAATCACGCTAAGCGT

C.brenneri GAAGCATTTATGGTTGAAATGTTTGAAGGATCTGTATTAATTTGTAACCACGCGAAGCGC

C.japonica GAGGCGTTCATCGTCGAGATGTTCGAGGGATCTGTTCTCATCTCGAATCACGCAAAACGG

PS_C.elegans GAAGCATTTTTGGTCGAAATGTTCGAAGGATCGTCTCTTATATCCACCCATGCGAAACGT

PS_C.remanei GAAGCGTTCATGGTGGAGATGTTCGAGGGATCCGTGTTGATTTGTAATCACGCGAAAAGA

PS_C.species9 GAAGCATTCATGGTTGAAATGTTCGAAGGATCCGTTCTGATTTGCAATCACGCTAAGCGT

PS_C.briggsae GAAGCATTCATGGTTGAAATGTTCGAAGGATCCGTACTGATTTGCAATCACGCTAAGCGT

PS_C.brenneri GAAGCATTTATGGTTGAAATGTTTGAAGGATCTGTATTAATTTGTAACCACGCGAAGCGC

PS_C.japonica GAGGCGTTCATCGTCGAGATGTTCGAGGGATCTGTTCTCATCTCGAATCACGCAAAACGG

C.elegans GTCACACTCATGACAACGGATATTCAGTTATACAGACGTCTCTGCCTTCGACATCTC---

C.remanei GTAACTCTCATGCCGACAGATATTCAATTATATCGTCGCTTATGTCTTCGGAATCTTTCA

C.species9 GTCACACTCATGCCCACTGACATTCAGCTGTATCGTCGCTTGTGCCTCCGAAACCTATCC

C.briggsae GTCACACTCATGCCCACTGACATTCAGCTGTATCGTCGCTTGTGCCTTCGAAACCTATCC

C.brenneri GTAACTCTTATGCCCACAGATATTCAATTATATCGACGTCTGTGCCTCAGAAATCTC---

C.japonica GTCACTCTGATGACGGCCGACATTCAATTGTACCGTCGACTTTGCCTCCGAAATCTC---

PS_C.elegans GTCACACTCATGACAACGGATATTCAGTTATACAGACGTCTCTGCCTTCGACATCTC---

PS_C.remanei GTAACTCTCATGCCGACAGATATTCAATTATATCGTCGCTTATGTCTTCGGAATCTT---

PS_C.species9 GTCACACTCATGCCCACTGACATTCAGCTGTATCGTCGCTTGTGCCTCCGAAACCTA---

PS_C.briggsae GTCACACTCATGCCCACTGACATTCAGCTGTATCGTCGCTTGTGCCTTCGAAACCTA---

PS_C.brenneri GTAACTCTTATGCCCACAGATATTCAATTATATCGACGTCTGTGCCTCAGAAATCTC---

PS_C.japonica GTCACTCTGATGACGGCCGACATTCAATTGTACCGTCGACTTTGCCTCCGAAATCTC---
