# Supplementary material for: Evidence for Centromere Drive in the Holocentric Chromosomes of Caenorhabditis
Source: PLoS One. 2012 Jan 23;7(1):e30496. doi: 10.1371/journal.pone.0030496 (PMC3264583; doi:10.1371/journal.pone.0030496)
Supplement: Text S2 — The alignment of CENP-CHCP-4 both prior and after the removal of unreliable regions. (DOC) [file pone.0030496.s004.doc]

C.japonica ATGAATCGAAAACCGTCC------TCGACAATCGTGCCCGGTCGACGCTACCCAACCGAA

C.briggsae ------------------------------------------------------------

C.remanei ATGGAACGAAAACATGGATGGCGCAGTACAATTGTTCCTGGCAGAAAAGCGATCACACAA

C.elegans ATGAATCGAAAACCGCGAACCCGCAGTACGATTGTTCCTGGTCGAAAAATGATCACTGAA

C.brenneri ATGGTCAATAAACGCTACGTTCGTAGTAGCATCGTTCCTGGTCGAAAAATGATCACAACA

PS_C.japonica ------------------------------------------------------------

PS_C.briggsae ------------------------------------------------------------

PS_C.remanei ------------------------------------------------------------

PS_C.elegans ------------------------------------------------------------

PS_C.brenneri ------------------------------------------------------------

C.japonica GCGGCAGCACTATGGGCTGCCGGCTACCGCGAAGATCCG---TCCTATTTGGCAGAAGTG

C.briggsae ------------------------------------ATG---TCATATTTAAATGAGAAA

C.remanei ATAGCTGCTTTACACGATGCCGGAGTTACCGAAGACATG---TCATACTTGAACGAAAAA

C.elegans ATCGTTGCTCTTCGTGAGGCTGGCTTGGACGATACAAGACCGTCGTACTTGGAGGAACCA

C.brenneri CAAGTAGCATTTCATCAAGCAGGAATCAGTGAAAACATT---TCATATTTAGCCGAAAAA

PS_C.japonica ------------------------------------CCG---TCCTATTTGGCAGAAGTG

PS_C.briggsae ------------------------------------ATG---TCATATTTAAATGAGAAA

PS_C.remanei ------------------------------------ATG---TCATACTTGAACGAAAAA

PS_C.elegans ------------------------------------AGA---TCGTACTTGGAGGAACCA

PS_C.brenneri ------------------------------------ATT---TCATATTTAGCCGAAAAA

C.japonica ACAGTGATGTCCGATAACACAATG---AACTCGACG---TGCGATGACGAGGAGAGACAA

C.briggsae AGCGTCTTGAACGAAACAACTCAACTGAATGAGACA---GGCGATGTTGAAGAACAAGTA

C.remanei TCCGTTTTGGATGAGTCGTCAAATTTGAATGATACTCATGATGACGACGAGGAAAGAGCA

C.elegans ACAGTTATCGTCGATGAATCGATGATGAATGATAGT---GCAAATTTGGAGGAGCGAGAA

C.brenneri ACCATAGTTGAAGATTCATCAATTCTCCACGAATCC---CATGACAACGAGGAAAGAGAA

PS_C.japonica ACAGTGATGTCCGATAACACAATG---AACTCGACG---TGCGATGACGAGGAGAGACAA

PS_C.briggsae AGCGTCTTGAACGAAACAACTCAA---AATGAGACA---GGCGATGTTGAAGAACAAGTA

PS_C.remanei TCCGTTTTGGATGAGTCGTCAAAT---AATGATACT---GATGACGACGAGGAAAGAGCA

PS_C.elegans ACAGTTATCGTCGATGAATCGATG---AATGATAGT---GCAAATTTGGAGGAGCGAGAA

PS_C.brenneri ACCATAGTTGAAGATTCATCAATT---CACGAATCC---CATGACAACGAGGAAAGAGAA

C.japonica TGGAAGCGTCAAGGGCTGTCGCAGAAGGAGATTTTTCAAATTCTAGAAGACAGA------

C.briggsae TGGAGAAAAGAAGGTTTGGATGAGAATGACATATTTAAACGACAGGAGGCTCGCAAACAG

C.remanei TTGAGAAAAAAAGGGTTCTCTGAGCGTGAAATTAACAAAAGACTGCAAGGTCAA------

C.elegans TGGAGAAAACAGGGACTATCTGAGAAGCAGATGTTCGCTATTTTGGAGAAAAGG------

C.brenneri TGGAAGAAACAAGGACTTTCCGATAGGGAAATTTATGACCGATTGCGAGATAAA------

PS_C.japonica TGGAAGCGTCAAGGGCTGTCGCAGAAGGAGATTTTTCAAATTCTAGAAGACAGA------

PS_C.briggsae TGGAGAAAAGAAGGTTTGGATGAGAATGACATATTTAAACGACAGGAGGCTCGC------

PS_C.remanei TTGAGAAAAAAAGGGTTCTCTGAGCGTGAAATTAACAAAAGACTGCAAGGTCAA------

PS_C.elegans TGGAGAAAACAGGGACTATCTGAGAAGCAGATGTTCGCTATTTTGGAGAAAAGG------

PS_C.brenneri TGGAAGAAACAAGGACTTTCCGATAGGGAAATTTATGACCGATTGCGAGATAAA------

C.japonica ------------------------AAGCTCAAGGAGATTCGAGATCACCAATCCCGTCTC

C.briggsae AAGCAGATGAGAGATGTGAGTTTACGAATCCAGTGGATGGAAAATGCAGAAGATCGTCTG

C.remanei ------------------------AGAGTTGAAAAACTCGCAAAGGCCCACGGTCGTCTC

C.elegans ------------------------AAACAAACACAGCTTAACAATGCCAGAAAACGTCTC

C.brenneri ------------------------AAACTAAAAAAGCTCAGAAATGCAGAGAACCGTCTA

PS_C.japonica ------------------------AAGCTCAAGGAGATTCGAGATCACCAATCCCGTCTC

PS_C.briggsae ------------------------CGAATCCAGTGGATGGAAAATGCAGAAGATCGTCTG

PS_C.remanei ------------------------AGAGTTGAAAAACTCGCAAAGGCCCACGGTCGTCTC

PS_C.elegans ------------------------AAACAAACACAGCTTAACAATGCCAGAAAACGTCTC

PS_C.brenneri ------------------------AAACTAAAAAAGCTCAGAAATGCAGAGAACCGTCTA

C.japonica GTCAAAGGTTACCGCGGCGCGAAAAACTACCACGAACTGCTCGCCGCCTACAATTGTCTT

C.briggsae AACAGAGAAAAATATGGAGCCAAGGGATTCGAGGACATGCTTGCCCTTCCACAATAT---

C.remanei GAAGAACAAATGTTAGGAGCGAAGACTTTCAAAGAATTGATGGAAAAACACGAGTAC---

C.elegans GAAAAGGAAATGTACGGAGCGAAAACCTTCAAAGAGTTGATTGCTTGTCGTGAATAC---

C.brenneri AACAAAGAGAAATTCGGTGCGAAAAATTTTATGGAATTACTGAACAAACATGAATAC---

PS_C.japonica GTCAAAGGTTACCGCGGCGCGAAAAACTACCACGAACTGCTCGCCGCCTACAATTGT---

PS_C.briggsae AACAGAGAAAAATATGGAGCCAAGGGATTCGAGGACATGCTTGCCCTTCCACAATAT---

PS_C.remanei GAAGAACAAATGTTAGGAGCGAAGACTTTCAAAGAATTGATGGAAAAACACGAGTAC---

PS_C.elegans GAAAAGGAAATGTACGGAGCGAAAACCTTCAAAGAGTTGATTGCTTGTCGTGAATAC---

PS_C.brenneri AACAAAGAGAAATTCGGTGCGAAAAATTTTATGGAATTACTGAACAAACATGAATAC---

C.japonica CCC---GATCAGCCGGGCCCTCCAGATGGTGACGTGGAGGCCGAAGAAAACGTGTCAAAT

C.briggsae TCTTCAGAGCGCGAGAGTGACGAAGACGACCACAACGAAGAAAATCAGAAAGCCCCCGAG

C.remanei TCA---GAACGAGAAAGCGACGATGAAAATGATACTGCTGAGAACATAACTATCACGAAT

C.elegans TCC---GAATGTGAGAGCGACAGCGAGAAC------ACTGTGAATCAAAATGTCCGAAGC

C.brenneri TCG---GAACGGGAAAGTGATGATGAAGAAAATGCTACTGTCGAAATGGATGTAAGAAAT

PS_C.japonica ------GATCAGCCGGGCCCTCCAGATGGT------GAGGCCGAAGAAAACGTGTCAAAT

PS_C.briggsae ------GAGCGCGAGAGTGACGAAGACGAC------GAAGAAAATCAGAAAGCCCCCGAG

PS_C.remanei ------GAACGAGAAAGCGACGATGAAAAT------GCTGAGAACATAACTATCACGAAT

PS_C.elegans ------GAATGTGAGAGCGACAGCGAGAAC------ACTGTGAATCAAAATGTCCGAAGC

PS_C.brenneri ------GAACGGGAAAGTGATGATGAAGAA------ACTGTCGAAATGGATGTAAGAAAT

C.japonica GCTCAGAAAAAGGACGACAGCGTGTTCGCCATCCCCAGCATGCCAAAAAAGAAGAAT---

C.briggsae ATT---------------CCAATATTTGCGATCCCTTCACTTCCGAAGCATATGAATGAA

C.remanei AAA---------------CCAGTTTTCGCTATTCCTGCACTTCCCAAGCATTTGAGTGAA

C.elegans GCT---------------TCGGTTTTTGCCATTCCGGCGCTTCCGAAACACATCAGTGAA

C.brenneri GTA---------------GAGGTTTTTGCAATCCCAGCGTTACCAAAACATTTGAGTGAG

PS_C.japonica GCT---------------AGCGTGTTCGCCATCCCCAGCATGCCAAAAAAGAAGAAT---

PS_C.briggsae ATT---------------CCAATATTTGCGATCCCTTCACTTCCGAAGCATATGAAT---

PS_C.remanei AAA---------------CCAGTTTTCGCTATTCCTGCACTTCCCAAGCATTTGAGT---

PS_C.elegans GCT---------------TCGGTTTTTGCCATTCCGGCGCTTCCGAAACACATCAGT---

PS_C.brenneri GTA---------------GAGGTTTTTGCAATCCCAGCGTTACCAAAACATTTGAGT---

C.japonica ------------------------------------------------GCAGGGCCGTCG

C.briggsae AAGTCAATGCTGGGTTCACCGGTTGCCGGTAGTAGAGGAAGCGGGAAGGCAGGTCTCAGT

C.remanei AAGTCAATGATGGGATCACCGGTTGCTGGAACGAAAGGAAGCGGAAAAGCCGGTCTCAGT

C.elegans AAGTCGATGATGGGCTCGCCAATTGCTAATTCCAGAGGGAGTGGAAAAGCTGGATTAAGT

C.brenneri AAGTCGATGTTCGGATCTCCGGTTTCGGGATCAAAAGGAAGCGGTAAAGCAGGCCTCAGT

PS_C.japonica ------------------------------------------------GCAGGGCCGTCG

PS_C.briggsae ------------------------------------------------GCAGGTCTCAGT

PS_C.remanei ------------------------------------------------GCCGGTCTCAGT

PS_C.elegans ------------------------------------------------GCTGGATTAAGT

PS_C.brenneri ------------------------------------------------GCAGGCCTCAGT

C.japonica TGCAGCACGCCGATCAGCGGAAAAGACGCATCGATGCGCACGCTCAAGGGACTGGACATT

C.briggsae TGTTCAACTCCAAAAAGTGCAAACGATGTCTCCATGCGTTCCCTTAGAGCCCTCGACCTG

C.remanei TGCTCAACTCCAAAAAGTGGAAAAGATGTTTCAATGCGTTCTCTAAGGGTTCTTGATATC

C.elegans TGTTCAACTCCCAAGAGCTCGAGTGATACGTCGATGAGGTCCTTGAGATCGCTTGATATT

C.brenneri TGCTCGACCCCAAAAAGTGCAAACGATTTGTCGATGCGTTCGTTGAGATCCTTAGATATT

PS_C.japonica TGCAGCACGCCGATCAGCGGAAAAGACGCATCGATGCGCACGCTCAAGGGACTGGACATT

PS_C.briggsae TGTTCAACTCCAAAAAGTGCAAACGATGTCTCCATGCGTTCCCTTAGAGCCCTCGACCTG

PS_C.remanei TGCTCAACTCCAAAAAGTGGAAAAGATGTTTCAATGCGTTCTCTAAGGGTTCTTGATATC

PS_C.elegans TGTTCAACTCCCAAGAGCTCGAGTGATACGTCGATGAGGTCCTTGAGATCGCTTGATATT

PS_C.brenneri TGCTCGACCCCAAAAAGTGCAAACGATTTGTCGATGCGTTCGTTGAGATCCTTAGATATT

C.japonica TCACAGGTTTTTTCA---------------------------------------------

C.briggsae TCGCATGTTATAAACACGGATCATCTTGATGCTAACAAA---------------------

C.remanei TCCCATGTCGTTAACACAGACCAATTAGATTATGACAAAGTGGCAGTCCATAACAAAAAC

C.elegans TCACATGTCGTCAATACCGATCGACTAGATGCTGAAAGAGTGACTGTTCACACCAAGTCT

C.brenneri TCCCATGTCGTTGCTGTAGACCAACTTGATGCGAACAAAGTGACAATTCAAAATAAAACC

PS_C.japonica TCACAGGTTTTTTCA---------------------------------------------

PS_C.briggsae TCGCATGTTATAAAC---------------------------------------------

PS_C.remanei TCCCATGTCGTTAAC---------------------------------------------

PS_C.elegans TCACATGTCGTCAAT---------------------------------------------

PS_C.brenneri TCCCATGTCGTTGCT---------------------------------------------

C.japonica ---------------------AACGTGGAATCGTCA------------------------

C.briggsae ------------------------------------------------------------

C.remanei GTACTTATTCCAATTGTGGAAAACACCGAACAATCT------------------------

C.elegans GTCGTTATCCCAACCATTCTCGAGGAGAGAGAGTCAACACTCCAGAAAACTCTCACGATT

C.brenneri ATTTTGCTTCCAATAGCGGAGAATGTTGAAAGTTCTTCGCTTCAAAAGACCCATACGATC

PS_C.japonica ------------------------------------------------------------

PS_C.briggsae ------------------------------------------------------------

PS_C.remanei ------------------------------------------------------------

PS_C.elegans ------------------------------------------------------------

PS_C.brenneri ------------------------------------------------------------

C.japonica ------------TTTACGACGGTAAAGTGTGCAGGAGGTGGTGGGGAACCGTCACTTCAG

C.briggsae ------------------------------------------------------------

C.remanei ------------TCGGCC---------------------------------AAAATGGGA

C.elegans GAGAAGAATCACAGCTCT---------------------------------CAGCTGCAA

C.brenneri CAG---------AGTTCA---------------------------------TCGCCACCG

PS_C.japonica ------------------------------------------------------------

PS_C.briggsae ------------------------------------------------------------

PS_C.remanei ------------------------------------------------------------

PS_C.elegans ------------------------------------------------------------

PS_C.brenneri ------------------------------------------------------------

C.japonica AAGACGTTTACG---ATGAGGAATGTGGAAGAGGAG------------------------

C.briggsae ------------------------------------------------------------

C.remanei GAAACTTTTACT---GTTAGAGATGACCAAGATGACAATCAAAAGCATGGACATGTATCT

C.elegans AAAACCTTCACTGTGGCTGAAGATGCTCCTGAAGAACTTCAGAAAACGGTGACAATCGAG

C.brenneri AAGACTTTCACT---GCTCAGGACTCATTG------CTTATGAAAACACATACGATTACA

PS_C.japonica ------------------------------------------------------------

PS_C.briggsae ------------------------------------------------------------

PS_C.remanei ------------------------------------------------------------

PS_C.elegans ------------------------------------------------------------

PS_C.brenneri ------------------------------------------------------------

C.japonica ---GAGCAGGAGTCG---ATTCAAAAAACTTTTACCGTCAGA---------AATGGCGAG

C.briggsae ------------------------------------------------ACGAATGGTGAA

C.remanei GCAAATGAAGCATCT---CTACAGAAAACGTTCACTGTAGATCCTAGAACAGAGGGCGAT

C.elegans AAGAATCAGAGCTCGGAAATGCAAAGAACGTTTACGGTGGCTAAAGATGCATCAAAAGAG

C.brenneri CGAGACTCGAACACCGAT---------------------TCAAACAACGAACGGGATGAA

PS_C.japonica ------------------------------------------------------------

PS_C.briggsae ------------------------------------------------------------

PS_C.remanei ------------------------------------------------------------

PS_C.elegans ------------------------------------------------------------

PS_C.brenneri ------------------------------------------------------------

C.japonica ------GAAGAGTCGCTGCAGAAGACGTTTGTGGTGGCGGAAGCCGACGGTGGCGCTTCT

C.briggsae ------GAATCAGATCTTCAAAAGACTCATACTATTCAAAAA------------ATGGCA

C.remanei AAATCAGAATCAGCTCTTCAGAAAACTTTCACTCTACATCCA------------AGAGCA

C.elegans ------CAATCACAGCTTCAAAGAACGGTAACGATCGAGAAG------------AATCAA

C.brenneri ------GAGTCTACCCTTCAAAAAACCCATACTATT---------------------CCG

PS_C.japonica ------------------------------------------------------------

PS_C.briggsae ------------------------------------------------------------

PS_C.remanei ------------------------------------------------------------

PS_C.elegans ------------------------------------------------------------

PS_C.brenneri ------------------------------------------------------------

C.japonica TCTTCTTCT---------------------------------------------------

C.briggsae AGTAGCGAA---------------------------------------------------

C.remanei GAGGGCGATAAATCA---------------------------------------------

C.elegans AGCTCTCAG------CTGCAAGGAAACTTCACGGTGGCTAAAGATGCTCCTGAAGAACTT

C.brenneri AGTTCTTCA------CTCCAGAAAAGTGAATCAATTCAAGATGGGTCA---------TTA

PS_C.japonica ------------------------------------------------------------

PS_C.briggsae ------------------------------------------------------------

PS_C.remanei ------------------------------------------------------------

PS_C.elegans ------------------------------------------------------------

PS_C.brenneri ------------------------------------------------------------

C.japonica ------------------------------TCGTCGTCGCTTCAGAAGACTTTTACCGTC

C.briggsae ------------------------------GGATCAACTCTTCAAAAGACTCATACTATT

C.remanei ------------------------------GAATCAAGTCTTCAGAAAACATTTACTGTT

C.elegans CAGAAAACGGTGACGATCGAGAAGAATCAGAGCTCGGAAATGCAGGGAACGGTCACAGTG

C.brenneri CAGAAAACTTACACTATT---------CAAGATGCCTCACTCCAAAAGACATTCACTGTT

PS_C.japonica ------------------------------------------------------------

PS_C.briggsae ------------------------------------------------------------

PS_C.remanei ------------------------------------------------------------

PS_C.elegans ------------------------------------------------------------

PS_C.brenneri ------------------------------------------------------------

C.japonica AGG---------------------------------------------------------

C.briggsae CAT---------------------------------------------------GAA---

C.remanei CCTGGAGGTGATCAGGATTCTACGAGACATTCCTCACTTCAAAATACTTTTGTAAAA---

C.elegans GCT------------AAAGATGCAGAACACTCCTCTCTACAGAAAACGTTCACAAAGCCG

C.brenneri CCT------------GATTCAACTGTTCATAAAAATGTTATCGACTCAAACACGTCGCCT

PS_C.japonica ------------------------------------------------------------

PS_C.briggsae ------------------------------------------------------------

PS_C.remanei ------------------------------------------------------------

PS_C.elegans ------------------------------------------------------------

PS_C.brenneri ------------------------------------------------------------

C.japonica ------------------------------------------------------------

C.briggsae ------------------------------------------------------------

C.remanei ------------------------------------------------------------

C.elegans ACCGAGAAAGATGAGTCA------------------------------------------

C.brenneri TCAAAAACCGATGATGCACAGGATAATCAAAGCTGCCAGCTACAGGAAGAAGGAAGTGAC

PS_C.japonica ------------------------------------------------------------

PS_C.briggsae ------------------------------------------------------------

PS_C.remanei ------------------------------------------------------------

PS_C.elegans ------------------------------------------------------------

PS_C.brenneri ------------------------------------------------------------

C.japonica ------------------------------------------AATGAAGAGGAGCCACTT

C.briggsae ------------------------------------GATGGAAGTAACGAAAGAAACTTA

C.remanei ------------------------------------AGCGGTTCGAACGATAGTTTACTG

C.elegans ---TCACTTCAAAGAACTTTCAACGTAGCTAACAGAGATGAAAATAACGATTCAACTCTT

C.brenneri CTGTCTGTTCAAAAGACTTTTGATGTT---GATGCTGATGAACAGAAC---AGTACATGT

PS_C.japonica ------------------------------------------------------------

PS_C.briggsae ------------------------------------------------------------

PS_C.remanei ------------------------------------------------------------

PS_C.elegans ------------------------------------------------------------

PS_C.brenneri ------------------------------------------------------------

C.japonica CAAGGTACATTTGTGGTGGAGAAG---------------------------------GAG

C.briggsae ------------------------------------------------------------

C.remanei ------------------------------------------------------------

C.elegans CAAAAGACGTTTATTATTGAAGAA---------------------------------AGA

C.brenneri GAAGGCACATTCGTAGTCCAGTCTGGATGGAGAAATACATTACTTGACTCGCTTCGACGA

PS_C.japonica ------------------------------------------------------------

PS_C.briggsae ------------------------------------------------------------

PS_C.remanei ------------------------------------------------------------

PS_C.elegans ------------------------------------------------------------

PS_C.brenneri ------------------------------------------------------------

C.japonica AAAGAGGCGGAGCCTGAGACT------------------------------GAG------

C.briggsae ---------------CCTACT------------------------------CCA------

C.remanei ---------------TCAAAT------------------------------AATGGGAAG

C.elegans GATGCATACGAGCAAGGAACC------------------------------ACG------

C.brenneri GATCAAGAAGCTCAGAGCTCGAACATAGGAAATGGAGATCTTTTGTCGCCATCT------

PS_C.japonica ------------------------------------------------------------

PS_C.briggsae ------------------------------------------------------------

PS_C.remanei ------------------------------------------------------------

PS_C.elegans ------------------------------------------------------------

PS_C.brenneri ------------------------------------------------------------

C.japonica ---------------------------CATAAACCCATTCCACAACCA------------

C.briggsae ---------------------------GAAGAAAACGATATCGCTGAC------------

C.remanei CGAGCTCAAGATTACACAAAGAAAATTGATGAAAGTGTCAATGCTGATCAATCAGGTTCT

C.elegans ---------------------------AGTGTTGGTGTGAAGCCTCCA------------

C.brenneri ---------------------------TCAACAGCGAATAATGTACCC------------

PS_C.japonica ------------------------------------------------------------

PS_C.briggsae ------------------------------------------------------------

PS_C.remanei ------------------------------------------------------------

PS_C.elegans ------------------------------------------------------------

PS_C.brenneri ------------------------------------------------------------

C.japonica ---------------------------------------------------------CAA

C.briggsae ---------------------------------------------------------ACC

C.remanei GTAATTGATTCTAGCAAACTGGCTGGCAACTGTATTTCGAACGAACTAATCGACAGAACA

C.elegans ---------------------------------------------------------GTA

C.brenneri ---------------------------------------------------------CCG

PS_C.japonica ------------------------------------------------------------

PS_C.briggsae ------------------------------------------------------------

PS_C.remanei ------------------------------------------------------------

PS_C.elegans ------------------------------------------------------------

PS_C.brenneri ------------------------------------------------------------

C.japonica GAACCCCCCAAAACC------------------CACAACCGCCCCAAAAAAACCAACCTT

C.briggsae GATGTTCGCAACCCTTCGAATGGAGGCGAACAGAAGAGAAAAGCAAAGAAAGTTGGGGCT

C.remanei ATCACGGAAAACACAGTTGAAGGAGGAGAACAAAGAAGACGATCAAATAAAGTTGGAGTT

C.elegans CTGGCACAAAACACGATGGAAGGAGGAGAAAAACGGAGAACGTCGAAAGTCACTTCG---

C.brenneri GTTTCACAAAACCAGCTTCAAGGAGGAGAGAAAAGGACGGGCCCGAAAAAAACAAGTCTA

PS_C.japonica ------------------------------------------CCC---------------

PS_C.briggsae ------------------------------------------GCA---------------

PS_C.remanei ------------------------------------------TCA---------------

PS_C.elegans ------------------------------------------TCG---------------

PS_C.brenneri ------------------------------------------CCG---------------

C.japonica GAAAAACGCGAAGGCCGCATGGCAGACCTCACCAGCTCAATGATGATGGGC---ATCGAT

C.briggsae CAAGACAGAGAAAGGCGTGGAGCAAATCTTAGTATGTCTCTGATGAATTCTATGATGGAA

C.remanei CAAGAGCGGGAAAGACGCCATGCAGATTTGAACTCTTCTTTGATGAAAAGTATGATTGAG

C.elegans GATGAGCGAGAACGACGTAATGCCAGTATTTCTAATCCTCTTCACAACAGTATGCTCGAA

C.brenneri CAAGAAAGAGAAAGGCGT------GCTGTTAACAGCTCTTTAATGAGTAGCATGATTGAA

PS_C.japonica GAAAAACGCGAAGGCCGC---------CTCACCAGCTCAATGATGATGGGC---ATCGAC

PS_C.briggsae CAAGACAGAGAAAGGCGT---------CTTAGTATGTCTCTGATGAATTCT---ATGGAT

PS_C.remanei CAAGAGCGGGAAAGACGC---------TTGAACTCTTCTTTGATGAAAAGT---ATTGAA

PS_C.elegans GATGAGCGAGAACGACGT---------ATTTCTAATCCTCTTCACAACAGT---CTCGAA

PS_C.brenneri CAAGAAAGAGAAAGGCGT---------GTTAACAGCTCTTTAATGAGTAGC---ATTGAC

C.japonica ------GACACACCATCGCCGGGAGCCCGCCACTTCAAGCCGAACGCGCGTAAAAAACTC

C.briggsae ------GATGTACCTTCTTCCGGCGCCAATTTATTGAAA---CATCCGCTGAAAAAAGTT

C.remanei ------GAAGTTCCGTCTCCTGGTGCTAACTACTTTAAG---AATCCCCGTAAGAAGCTA

C.elegans AAGGAAGAAATTCAATCGCCGGGAGCCAACTTCTTCAAA---CATCCCCGAAAAAAGATC

C.brenneri ------GACATCCCGTCTCCTGGCGCTGGACTTTTCAAA---AATACTCGTAAAAAGTTG

PS_C.japonica ---------ACACCATCGCCGGGAGCCCGCCACTTCAAG---AACGCGCGTAAAAAACTC

PS_C.briggsae ---------GTACCTTCTTCCGGCGCCAATTTATTGAAA---CATCCGCTGAAAAAAGTT

PS_C.remanei ---------GTTCCGTCTCCTGGTGCTAACTACTTTAAG---AATCCCCGTAAGAAGCTA

PS_C.elegans ---------ATTCAATCGCCGGGAGCCAACTTCTTCAAA---CATCCCCGAAAAAAGATC

PS_C.brenneri ---------ATCCCGTCTCCTGGCGCTGGACTTTTCAAA---AATACTCGTAAAAAGTTG

C.japonica CGTGAATCGCTTCAAACACCGCCACGT---GGCCTGTCGAATCGAATGAGCCTCGATTCG

C.briggsae AGGCCGGAAACGAAGAGCCCACAAAAGCCACGAGTTATGGGTAGACTCAGTACAGAATCA

C.remanei AGACCTACTGTTGAAGTCCCG------CCAAAGATCATCAGCAGACTGAGTGTTGAATCA

C.elegans AGACCGGTGGTTCAAACGCCGCCACGAATCAAGGCAACCAGCAGACTTAGTACTGAATCG

C.brenneri AGACCAACAAACGTAACCCCACAAAGGATGAAG---ACCAACCGGTTAAGCACAGAGTCA

PS_C.japonica CGTGAATCGCTTCAAACACCG---------------TCGAATCGAATGAGCCTCGATTCG

PS_C.briggsae AGGCCGGAAACGAAGAGCCCA---------------ATGGGTAGACTCAGTACAGAATCA

PS_C.remanei AGACCTACTGTTGAAGTCCCG---------------ATCAGCAGACTGAGTGTTGAATCA

PS_C.elegans AGACCGGTGGTTCAAACGCCG---------------ACCAGCAGACTTAGTACTGAATCG

PS_C.brenneri AGACCAACAAACGTAACCCCA---------------ACCAACCGGTTAAGCACAGAGTCA

C.japonica GACAAGGACAAGACGATTTCGATGCTCTCGGTGGCCGAA------TCGTCGGGCCGCGAG

C.briggsae GACAAAGAGAAGACTATTGAGATGGGATCGATTGCTGAAGAGTCTTCAATA---GCTGAA

C.remanei GACAAAGAAAAAACTATTGAAATGTTGTCGATGGTCGAAGAAGTCTCAATGGAAGCCGAG

C.elegans AATAAGGAGCGAACGATTGAGATGGAATCGGTAGCTGAAGAACGAACAATGGAAGCGGAT

C.brenneri GATAAAGAAAAAACGATTGATATGCTGTCTATAGCTGAAGAACTTTCTATGGAAAATGAA

PS_C.japonica GACAAGGACAAGACGATTTCGATGCTCTCGGTGGCCGAA------TCGTCG---CGCGAG

PS_C.briggsae GACAAAGAGAAGACTATTGAGATGGGATCGATTGCTGAA------TCAATA---GCTGAA

PS_C.remanei GACAAAGAAAAAACTATTGAAATGTTGTCGATGGTCGAA------TCAATG---GCCGAG

PS_C.elegans AATAAGGAGCGAACGATTGAGATGGAATCGGTAGCTGAA------ACAATG---GCGGAT

PS_C.brenneri GATAAAGAAAAAACGATTGATATGCTGTCTATAGCTGAA------TCTATG---AATGAA

C.japonica TCGATGGGGTCGTCGTACGCCGATCCGGTGTCGATCCATCATCAGACGTCGCACATTTCG

C.briggsae TCGATGGGATCCTCATATGTAGATCCTGTTCCAGAGAAC------GCAACTGCTTTTTCA

C.remanei TCCAACGGTCCATCATTTGTTGATCCACTATCTGTGAAC------GGGTCTCGCATCTCA

C.elegans TCTATTGGCTCGTCTTATATCTCTCCA---ACATTCAAT------GCATCTCGAGTTTCG

C.brenneri ACAAATGGCTCCTCTTTCGTTGATCCGGTTTCTGTAAAT------GGGTCGCGAGTTTCG

PS_C.japonica TCGATGGGGTCGTCGTACGCCGATCCG---TCGATCCAT------ACGTCGCACATTTCG

PS_C.briggsae TCGATGGGATCCTCATATGTAGATCCT---CCAGAGAAC------GCAACTGCTTTTTCA

PS_C.remanei TCCAACGGTCCATCATTTGTTGATCCA---TCTGTGAAC------GGGTCTCGCATCTCA

PS_C.elegans TCTATTGGCTCGTCTTATATCTCTCCA---ACATTCAAT------GCATCTCGAGTTTCG

PS_C.brenneri ACAAATGGCTCCTCTTTCGTTGATCCG---TCTGTAAAT------GGGTCGCGAGTTTCG

C.japonica TCGATTCCGGAAGAGCACGAGGAGGAGCAGGAGGAGGTGGAAGTGGAA---------GAA

C.briggsae CCAGTTCCT------------------------------GAAGAAGAA---------GAA

C.remanei CCAATTCCA------------------------------GAAGTAGAC---------AAA

C.elegans CCGGTTCCA------------------------------GAAGTTCCAGAAAAAGTGGAG

C.brenneri CCTGTAATA------------------------------GAAGTTGAC---------GAG

PS_C.japonica TCGATTCCG---------------------------------GTGGAA------------

PS_C.briggsae CCAGTTCCT---------------------------------GAAGAA------------

PS_C.remanei CCAATTCCA---------------------------------GTAGAC------------

PS_C.elegans CCGGTTCCA---------------------------------GTTCCA------------

PS_C.brenneri CCTGTAATA---------------------------------GTTGAC------------

C.japonica GAGGCGGAGACGACTGTGCAGAATTCGGAGGCTTCAGTAGCCATAAATCAATTAAATACC

C.briggsae CCGATG------------------------------------------------------

C.remanei CTCACA------------------------------------------------------

C.elegans CCAATG------------------------------------------------------

C.brenneri CCAATG------------------------------------------------------

PS_C.japonica ------------------------------------------------------------

PS_C.briggsae ------------------------------------------------------------

PS_C.remanei ------------------------------------------------------------

PS_C.elegans ------------------------------------------------------------

PS_C.brenneri ------------------------------------------------------------

C.japonica AAAAAATCGCCCTTTCCTCACATTTCTCCTTCCAGAACCCCTCCACGAGCTCCCAGACTC

C.briggsae ------------------GAGATTGCGAAC------ACCACTCCCAAATCAACCCGCCGT

C.remanei ------------------AATCCGACGAAT------GTCACACCAAAATCAAATCGTCGC

C.elegans ------------------CATGTATCGAAA---GCCGCAACTCCAAAATCGATTCGGCAC

C.brenneri ------------------GATATTTCGAAAAATGAAACAACTCCAAAATCAAGGAGGCAT

PS_C.japonica ------------------------------------------------------------

PS_C.briggsae ------------------------------------------------------------

PS_C.remanei ------------------------------------------------------------

PS_C.elegans ------------------------------------------------------------

PS_C.brenneri ------------------------------------------------------------

C.japonica ---------------ACA------CAATCGTCGATCGAACGT------------------

C.briggsae GGATCAAGCTTCGTAACT------CCCCACAGTTTG---ATG------------------

C.remanei AATCTTCCCATC------------------------------------------------

C.elegans ---------------ACG------GATAATTCTATC---CGTAATATTGGACCCACCAAC

C.brenneri ---------------ACTCTTCTCAACAGCAATTTG---AGA------------------

PS_C.japonica ------------------------------------------------------------

PS_C.briggsae ------------------------------------------------------------

PS_C.remanei ------------------------------------------------------------

PS_C.elegans ------------------------------------------------------------

PS_C.brenneri ------------------------------------------------------------

C.japonica ------CGCCGAGCCAACCAATCGGCTTTACGCATTTCC------ACTGCACCGAAAGCC

C.briggsae ------GAAAGAGCAAGAGGAGGGACACTTTTCTCAACT------CCAGTTCCACCAGTT

C.remanei ---------------CGAGGCAATTCTATGGAAACCGTG------CGAGTAGCTCAGGCA

C.elegans AATGTCGAATGTGCTCGGGCTGCACTGCTCTCAACACCG------ACTAGGATGGATATC

C.brenneri ------GAAAGGGCCCGGGCTCTAAGTATTTCGGCTAGTCCTGCTAGCGCTATGAAAGTA

PS_C.japonica ------------------------GCTTTACGCATTTCC---------------------

PS_C.briggsae ------------------------ACACTTTTCTCAACT---------------------

PS_C.remanei ------------------------TCTATGGAAACCGTG---------------------

PS_C.elegans ------------------------CTGCTCTCAACACCG---------------------

PS_C.brenneri ------------------------AGTATTTCGGCTAGT---------------------

C.japonica GCTGAA------GGAACCTTTCTGACCACGCCCACCGCTCACAATTACCAAAAACCGACT

C.briggsae GTTGAC------GCC---------GTCACACCCAAA---CTTAACTATCAGAAACAAACC

C.remanei AATGAT------GTTTCTTCTGTCATTACTCCCAAG---CTCAATTATCTGAAACCTACT

C.elegans GTTGAT------TCTGTGAATCGAGTGACCCCGGCA---CGACGATATGAACAGCCTACT

C.brenneri GTAGAGGACAACGCTCCGACTGACATTACTCCCAAA---CGTCATTATCTAACCCCTACG

PS_C.japonica ------------------------ACCACGCCCACC---CACAATTACCAAAAACCGACT

PS_C.briggsae ------------------------GTCACACCCAAA---CTTAACTATCAGAAACAAACC

PS_C.remanei ------------------------ATTACTCCCAAG---CTCAATTATCTGAAACCTACT

PS_C.elegans ------------------------GTGACCCCGGCA---CGACGATATGAACAGCCTACT

PS_C.brenneri ------------------------ATTACTCCCAAA---CGTCATTATCTAACCCCTACG

C.japonica TTCTCTTCGTTGGTGAAAAGCAAGGACCGAGCCGAGTGCACCGAGTTGCTCACA------

C.briggsae GTTTCATCAGCATTAAAAATGAAGGGTGCTCCCAATAGCTGTGAACTTCTTGAT------

C.remanei ATATCATCATTGAGAAAACATGTTAATGAACCCGAATGCGACGATACCCTTTTC------

C.elegans TTCGCTTCACTCGTCAAAAGGATGAATATGAAAGATGCTAATCGTTTGCTTGAG------

C.brenneri TTTTCTTCATTAGTGAAGAAAAAGACAGCTGTTGAGATCAATGATCTACTGGAAGCAAAG

PS_C.japonica TTCTCTTCGTTGGTGAAAAGCAAGGACCGAGCCGAGTGCACCGAGTTGCTCACA------

PS_C.briggsae GTTTCATCAGCATTAAAAATGAAGGGTGCTCCCAATAGCTGTGAACTTCTTGAT------

PS_C.remanei ATATCATCATTGAGAAAACATGTTAATGAACCCGAATGCGACGATACCCTTTTC------

PS_C.elegans TTCGCTTCACTCGTCAAAAGGATGAATATGAAAGATGCTAATCGTTTGCTTGAG------

PS_C.brenneri TTTTCTTCATTAGTGAAGAAAAAGACAGCTGTTGAGATCAATGATCTACTGGAA------

C.japonica GAGTTGAATAAA---AATCGAACGCCGAGACGAACAGTGCCGCCAGTGGAAGAGCCGGCT

C.briggsae GTGGATCGTAGATGTCGCTCAGGTCCGAAGAAATCCGTCAATGTGGCCAGAGAGTCACCG

C.remanei GGTACTCGTAGAGACCGATGCACACCTGGAAAAAGTGCCACGACGGCAAAAACAGTGGTT

C.elegans GAAACTTCACGA---AAGAATACGCCTGCGAAGACTACTGCTACAACTTCTTCAGCCGCT

C.brenneri AAAAAAGATCGT---CGTAACACACCACATCGGAATGCTACGATTCAAGAAGAAGAAGCG

PS_C.japonica ---------------------ACGCCGAGACGAACAGTGCCGCCA---------------

PS_C.briggsae ---------------------GGTCCGAAGAAATCCGTCAATGTG---------------

PS_C.remanei ---------------------ACACCTGGAAAAAGTGCCACGACG---------------

PS_C.elegans ---------------------ACGCCTGCGAAGACTACTGCTACA---------------

PS_C.brenneri ---------------------ACACCACATCGGAATGCTACGATT---------------

C.japonica ---------CAGGAGCTTGGACACGTGGCAGAA---------------------------

C.briggsae ---------AAAGATAACGATGATGGGAATGGAAAACCG---------GAAGACGTTGTT

C.remanei ---------CAAGATGCACCGATCATTGAGAAGACGCCGGTAACTGGAGAAGGAGTAACA

C.elegans GTTCGCATGGTTTTGGAGGATGATGAGGAGGATCAGGCG---------ACGGAGGTCATT

C.brenneri GTATCATTAAGTGCTGGCGAAGGAGTAGACGACATCCAC---------AGTGAATCTGTT

PS_C.japonica ------------------------------------------------------------

PS_C.briggsae ------------------------------------------------------------

PS_C.remanei ------------------------------------------------------------

PS_C.elegans ------------------------------------------------------------

PS_C.brenneri ------------------------------------------------------------

C.japonica ---------------------------------------------GACCTGGAAGCTCTT

C.briggsae ATTGATGATAGCAAA------------------------TTGAATGATGAAGAAATGTGT

C.remanei GTCAAAAATGATCAG------------------------CGAAATGATGCGAGCAACAGA

C.elegans GAAAAAAGATCTGAAAATGGTGGTGTGATTGTCGATGGTGAGGATGAGGCAGCA------

C.brenneri CCCCTTAGCCAACAA------------------------GTGGATGACTGTGCT------

PS_C.japonica ------------------------------------------------------------

PS_C.briggsae ------------------------------------------------------------

PS_C.remanei ------------------------------------------------------------

PS_C.elegans ------------------------------------------------------------

PS_C.brenneri ------------------------------------------------------------

C.japonica TCGATCAGTGTTGGGGTGCTGGAGAAGGAGCAGCAGGAGCCCAGACCACTG---------

C.briggsae GATGTGACGGTTGAAATGCCCCAAAATGTCGAACAGGCAGCGAGCGGTGTAGAACTC---

C.remanei GATCTTTCCACCGGATTGCCCGAAGAGACCAATCGACCGTCGTTCAATCTTGAGTTA---

C.elegans ------------------------------GATTCAAGCAATCGCTCCCTGAACATC---

C.brenneri ------------------------------GAAAAATCTTCCAAGCCAGTTGATATTAGT

PS_C.japonica ------------------------------------------------------------

PS_C.briggsae ------------------------------------------------------------

PS_C.remanei ------------------------------------------------------------

PS_C.elegans ------------------------------------------------------------

PS_C.brenneri ------------------------------------------------------------

C.japonica ------------------------------------------------------------

C.briggsae ---------------------------GATATGAACGGATTGACACTTCATTCAGCT---

C.remanei ---------------------------GAAGTGGGTGATATGTCAATCCGGCCGTCTCCT

C.elegans ---------------------------GAATTGAATGCGCTGACTGTCAACGAAGAGCCT

C.brenneri AGTCATTCAATTACAAGCATAAGAATGGAGTTAGATGAAATGACTGTTAATGATGGCCCG

PS_C.japonica ------------------------------------------------------------

PS_C.briggsae ------------------------------------------------------------

PS_C.remanei ------------------------------------------------------------

PS_C.elegans ------------------------------------------------------------

PS_C.brenneri ------------------------------------------------------------

C.japonica ---------------------------------ACAGAAGACGTCGATTTC---------

C.briggsae ---AAC------GTAAGCTATAACTTGGACCATGACGGTTTGGATGATTTCCATGGA---

C.remanei AAAAGA------TTGAGTGCTAACTTGGATTCGGTGGAACCTGCTGATTTT---------

C.elegans GCACAT------GATATCAGTGCCATTGATTTTCCAGAGGACGATACAAAT---------

C.brenneri TCCATGGTCATAGACTACGATGCAGTCGATTATGTAGATCATAATGATCGT------TCC

PS_C.japonica ------------------------------------------------------------

PS_C.briggsae ------------------------------------------------------------

PS_C.remanei ------------------------------------------------------------

PS_C.elegans ------------------------------------------------------------

PS_C.brenneri ------------------------------------------------------------

C.japonica ---------------GAA---------------GATCGAGAAGAGCCAGTT---CCAGAC

C.briggsae ---------------GATCCCAATTTTGAAGATGAAAGAAACGAAAGTGAGGATGCTGGG

C.remanei ---------------GACTTGGACCTCCCAACTGTGAGAATCGAGAATCAG---GCGGGT

C.elegans ---------------GAAATGAGATCTTCTTCTGACGAAGAAGAAATGGAA---GCACGT

C.brenneri GATCAGTTTGATGCTGAAAGTCTGGAAGAGTCGGAAGGAGAAAGTGATGAG---GCCGGG

PS_C.japonica ------------------------------------------------------------

PS_C.briggsae ------------------------------------------------------------

PS_C.remanei ------------------------------------------------------------

PS_C.elegans ------------------------------------------------------------

PS_C.brenneri ------------------------------------------------------------

C.japonica GACGAGTCGGTCAAAAAGATGCTCAGAAGA---GTCGGATTGCTCTCTGACTCTATCGCC

C.briggsae TCTTCTACCCGCAGAACAACTAGAAGTCGA---ATTGGATTACTCAGTGACTCAATCGCT

C.remanei CCTTCTAATCAAAGATCATCCAGAAATAGA---GCTGCTCTGCTTAGTGACTCGATTGCC

C.elegans CCAGATCCAAGGAAAAAGTCGTCTAGAAGA---CTCGGGCTTCTTAGTGATTCGATTGCT

C.brenneri CCCTCCTCGAGAAAGAAATCTTCACGGCGAAAGATTGGAACGTTGAGCGATTCAATGACA

PS_C.japonica ---------------------------------GTCGGATTGCTCTCTGACTCTATCGCC

PS_C.briggsae ---------------------------------ATTGGATTACTCAGTGACTCAATCGCT

PS_C.remanei ---------------------------------GCTGCTCTGCTTAGTGACTCGATTGCC

PS_C.elegans ---------------------------------CTCGGGCTTCTTAGTGATTCGATTGCT

PS_C.brenneri ---------------------------------ATTGGAACGTTGAGCGATTCAATGACA

C.japonica ACTGTAAACACCCCTGGACATCCTAGAGCCGCTGTT---------CATTTC---------

C.briggsae ACTGTGAATTCGCCTGGCGTCGATCGTCGTCAAACTGGGAAAAATTACAGG---------

C.remanei ACTGTGAACACTCCGGGGTATAACCGTACTGCTAGATGTCGT---GTCATG---------

C.elegans CTTGGTCTTGCT---------------AGTTCGTCGAGAAGA---CGCCCA---------

C.brenneri TCCATGGATGCT---------------CGGATAAGTGGAAAC---CATTCTAATTTTGCA

PS_C.japonica ------------------------------------------------------------

PS_C.briggsae ------------------------------------------------------------

PS_C.remanei ------------------------------------------------------------

PS_C.elegans ------------------------------------------------------------

PS_C.brenneri ------------------------------------------------------------

C.japonica AACGACACCACTGCAGTGGATCAGTGGAGATCCGATGAGGATGATGACGAGGAAGAAGAA

C.briggsae AATGATACTATTCCAGAAGACTCGTGGGATTCTGATGAA---GAAGTCGTT---------

C.remanei AATAACACGAATGTGGAAGAATCGTGGCAATCCGATGAAGACGACGTCATT---------

C.elegans AACGACACATTCGTTGACGAAACATGGTATCCT---GAA---------------------

C.brenneri AATGATACTATCTCTAACGAAACATGGCAACCAGGGGAA---------------------

PS_C.japonica ---GACACCACTGCAGTGGATCAGTGGAGATCC---------------------------

PS_C.briggsae ---GATACTATTCCAGAAGACTCGTGGGATTCT---------------------------

PS_C.remanei ---AACACGAATGTGGAAGAATCGTGGCAATCC---------------------------

PS_C.elegans ---GACACATTCGTTGACGAAACATGGTATCCT---------------------------

PS_C.brenneri ---GATACTATCTCTAACGAAACATGGCAACCA---------------------------

C.japonica GAAGAAGGCCCATCCAGAAGACGTCAAACGAGACGAGGAGGAAAGAAACAAGAAGTCGGA

C.briggsae ------------TCTAGAAGGCGCAATGATGTTAGAAACACTGTAAAA------ATTGGA

C.remanei ------------ATCAGTAGGAGAAATCCGGGGAAAAAT---------------GTTGGA

C.elegans ------------CCGAATTCTAAAGGAAACAGACGTCCGAGAACACAC------AGGGGT

C.brenneri ------------ACTTCCAGAAAAAATAACCGAGGAGGGAGGAACAAA------ACCTCA

PS_C.japonica ------------------------------------------------------------

PS_C.briggsae ------------------------------------------------------------

PS_C.remanei ------------------------------------------------------------

PS_C.elegans ------------------------------------------------------------

PS_C.brenneri ------------------------------------------------------------

C.japonica ---CTGCAATTGGCGAAACGGAGAATTATCGAGCCCGAACAAGCGCCCGACGGCATCCGA

C.briggsae ---TTGCAGCTAAAGAAGAGAGAAATTATTCAACCTGACGATGCAACGAATGGTGTTAGA

C.remanei ---CTCCAGCTCAAAAAGCGCGAAATAATTCAACCA---GACACAAATAATGGAAACAGA

C.elegans ---ATGAAACTGAAAGAGCATCAACTCATGAAGCCAGAAGATGCTCCAGACGGTGTCCGA

C.brenneri GAAATGCAGCTCAAGAAACGAGAAATCATTCAACCTGCCTCTCCACAGGGCCAAGTCCGG

PS_C.japonica ---CTGCAATTGGCGAAACGGAGAATTATCGAGCCC------GCGCCCGACGGCATCCGA

PS_C.briggsae ---TTGCAGCTAAAGAAGAGAGAAATTATTCAACCT------GCAACGAATGGTGTTAGA

PS_C.remanei ---CTCCAGCTCAAAAAGCGCGAAATAATTCAACCA------ACAAATAATGGAAACAGA

PS_C.elegans ---ATGAAACTGAAAGAGCATCAACTCATGAAGCCA------GCTCCAGACGGTGTCCGA

PS_C.brenneri ---ATGCAGCTCAAGAAACGAGAAATCATTCAACCT------CCACAGGGCCAAGTCCGG

C.japonica CGTTCGAGCAGAGTCCGCGTGAAACCTCTCCGCTCTTGGCTCGGCGAACGCCTTGACTAC

C.briggsae CGATCTCAACGCAATAGAGTCAAGCCTGTTCGGTCGTGGCTTGGAGAGAAACCTGTGTAC

C.remanei CGATCAACACGCAACAGAGTTAAACCACTCCGGTCCTGGCTTGGAGAGAAAGCCGTGTAC

C.elegans AGATCGACGCGTGTCCGCGTGAAGCCTGTTCGTTCGTGGCTTGGAGAACAACCGGTTTAT

C.brenneri CGATCAGAACGTGTTCGTGTGAAACCTGTTCGATCTTGGCTAGGTGAAAAAGCGGTGTAC

PS_C.japonica CGTTCGAGCAGAGTCCGCGTGAAACCTCTCCGCTCTTGGCTCGGCGAACGCCTTGACTAC

PS_C.briggsae CGATCTCAACGCAATAGAGTCAAGCCTGTTCGGTCGTGGCTTGGAGAGAAACCTGTGTAC

PS_C.remanei CGATCAACACGCAACAGAGTTAAACCACTCCGGTCCTGGCTTGGAGAGAAAGCCGTGTAC

PS_C.elegans AGATCGACGCGTGTCCGCGTGAAGCCTGTTCGTTCGTGGCTTGGAGAACAACCGGTTTAT

PS_C.brenneri CGATCAGAACGTGTTCGTGTGAAACCTGTTCGATCTTGGCTAGGTGAAAAAGCGGTGTAC

C.japonica GCATTCTCGCCG---AACGGTACACGTCGTCTTAAAGGCGTCAACGACGTCTTCATTAAG

C.briggsae GTGAACTCTCCA---AGCGGAGGAAAGAGACTGACTGGAGTTACTGATGTTATTATCAAA

C.remanei AAAAACTCGCCA---AGTGGAGGAAAACGTTTAACTGGCGTGACTGATGTCGTTATCAAA

C.elegans GTCAATTCGCCGATTAGTGGCTGCAAGCGATTGACTGGTGTGACGGCCGTTGTCATCAAG

C.brenneri GTGAACTCACCA---AGGGGAGGAAAGCGTTTGACTGGCGTCACAGATGTCATCATCAGG

PS_C.japonica GCATTCTCGCCG---AACGGTACACGTCGTCTTAAAGGCGTCAACGACGTCTTCATTAAG

PS_C.briggsae GTGAACTCTCCA---AGCGGAGGAAAGAGACTGACTGGAGTTACTGATGTTATTATCAAA

PS_C.remanei AAAAACTCGCCA---AGTGGAGGAAAACGTTTAACTGGCGTGACTGATGTCGTTATCAAA

PS_C.elegans GTCAATTCGCCG---AGTGGCTGCAAGCGATTGACTGGTGTGACGGCCGTTGTCATCAAG

PS_C.brenneri GTGAACTCACCA---AGGGGAGGAAAGCGTTTGACTGGCGTCACAGATGTCATCATCAGG

C.japonica GACAAACGAATGTGCAAATACCGTACCGCCGACTGCCGGCTCGCCATGGAAAGAGAGCAA

C.briggsae GACAAACGATTGTGCAAGTACAGAACTGGGGACTCGTTC---------------------

C.remanei GACAAAAGACTGTGCAAATATCGGACCGCTGATCTTAAACTGGCTACTGAACGAGAACAG

C.elegans GATCCGCGTTTGTGCTATTACAGAACTGCTGACGTCAGAACAGCAACCGAGCGAGAGCTG

C.brenneri GACAAGCGTTTGTGCAAGTACCGAACCGCTGATCTCAAACTAGCTACGGAGCGAGAGCAA

PS_C.japonica GACAAACGAATGTGCAAATACCGTACCGCCGAC---------------------------

PS_C.briggsae GACAAACGATTGTGCAAGTACAGAACTGGGGAC---------------------------

PS_C.remanei GACAAAAGACTGTGCAAATATCGGACCGCTGAT---------------------------

PS_C.elegans GATCCGCGTTTGTGCTATTACAGAACTGCTGAC---------------------------

PS_C.brenneri GACAAGCGTTTGTGCAAGTACCGAACCGCTGAT---------------------------

C.japonica CGGGAAAAGGCGCGAAAGAGGGAGAGAGCGGCGAAGAAAAGGGCACGATTGGCGTTGGAT

C.briggsae ------------------------------------------------------------

C.remanei CGAGCAAAGGCACACAAAAAGGAATTGGCTGCTAGGAAACGAGAACAGCTGCTCAGAGAT

C.elegans AAAGATAAGGCCAACAAAAGGGCTTTGGCGCAGGAAAAG---AAGCAACAAAGACAGAAT

C.brenneri AAAGAGAAGGCTTACAAAAAGAGAGTTGCCGCTGAAAAACGTAAAAAATTAGCTGCAGAT

PS_C.japonica ------------------------------------------------------------

PS_C.briggsae ------------------------------------------------------------

PS_C.remanei ------------------------------------------------------------

PS_C.elegans ------------------------------------------------------------

PS_C.brenneri ------------------------------------------------------------

C.japonica CAGAGCCAGGGACGTCGGATGGACGAGTCGCAGGAGGATATCGTCACGTCTTCTGATGAG

C.briggsae ------------------------------------------------------------

C.remanei CAACAAGCAGGGCGCCGAATGGACGAATCACATTATGATATTCATACCGATGACGAAGAA

C.elegans GCAAGAAGTGGGCGACGT---CACGATAGTGACGACGAT---------GAAGAAGATGAT

C.brenneri CAAAGAAGAGGGCGTCGTTTAAATGAATCCCAGGATGATATTTTCACCGATGATGATGAC

PS_C.japonica ------------------------------------------------------------

PS_C.briggsae ------------------------------------------------------------

PS_C.remanei ------------------------------------------------------------

PS_C.elegans ------------------------------------------------------------

PS_C.brenneri ------------------------------------------------------------

C.japonica GAG

C.briggsae ---

C.remanei ---

C.elegans ATG

C.brenneri CAA

PS_C.japonica ---

PS_C.briggsae ---

PS_C.remanei ---

PS_C.elegans ---

PS_C.brenneri ---
