# Supplementary material for: Correction: Evidence for Centromere Drive in the Holocentric Chromosomes of Caenorhabditis
Source: PLoS One. 2016 Jan 26;11(1):e0147889. doi: 10.1371/journal.pone.0147889 (PMC4728075; doi:10.1371/journal.pone.0147889)
Supplement: S1 Text — (DOC) [file pone.0147889.s001.doc]

>C.elegans

ATGGCCGAT------------------GACACCCCAATTATTGAGGAAATCGCCGAGCAA

AATGAGAGCGTCACAAGGATCATGCAACGTCTC------------------------AAA

CATGAC---------------------------ATGCAAAGAGTCACTTCAGTGCCGGGA

------TTCAACACAAGCGCCGCAGGTGTCAACGATTTGATCGACATTCTG---AACCAG

TATAAGAAGGAGCTTGAGGATGATGCAGCCAACGACTACACTGAAGCGCACATCCACAAA

ATTCGATTGGTCACAGGCAAACGGAATCAA------------------------TATGTC

TTGAAGTTG------------------------AAGCAAGCCGAAGACGAATATCACGCG

CGAAAA---------------------------------GAGCAAGCTCGGAGAAGAGCT

------------------------TCGTCTATGGATTTCACGGTCGGCAGAAATTCCACG

AATCTTGTC---------GAT------------------TACTCCCACGGCCGTCATCAT

ATGCCCTCATACCGT---------CGACACGATAGCTCCGACGAAGAA---AACTAT---

------------------TCTATGGATGGAACAAATGGCGAT------------------

---GGAAATAGAGCTGGC------------------------------------------

CCATCGAACCCC------------------------------------GAT---------

---------CGTGGTAAT------------------------------------------

------------------------------AGAACTGGC---------------CCATCG

AGCTCCGATCGCGTGCGGATGAGAGCCGGAAGGAACAGAGTCACCAAAACG------AGA

CGTTATAGACCGGGCCAGAAGGCATTGGAAGAGATCCGCAAGTACCAAAAAACTGAAGAC

CTTCTGATTCAAAAGGCTCCGTTCGCACGCCTCGTCCGCGAAATTATGCAGACTTCCACT

CCATTTGGCGCCGACTGCCGTATTCGTTCTGACGCCATCAGTGCTCTTCAAGAAGCGGCG

GAAGCATTTTTGGTCGAAATGTTCGAAGGATCGTCTCTTATATCCACCCATGCGAAACGT

GTCACACTCATGACAACGGATATTCAGTTATACAGACGTCTCTGCCTTCGACATCTC---

>C.remanei

ATGCACCAC------------------AATGGACCGCGTATTGAAGAAATGGTGGATCCA

CCG---TCCAGGAGCACAACAAACCAG---TTA------------------------AAA

AATGATACAGAGTATATCAAA------TCAGAATATCGCAGAATCAGCCATCTTCCAGAC

------TTCAACAGAGACCCAGAATTAATCCAGGAGGTTATGAATTTGACAAAAAGATAC

ATCGAAAAGTGGCTACGA---GAAGAAAGAGACGAACCGAATATGGAGCGTCAAGGATGG

ATTGAGCGCTTCAAAACAAAGCTTCGTGAA------------------------TGGGAA

ACGAAAAAA------------------------GAAACCGCAGAAGATGAATACTATACA

CGACGAGAC---------------------GCGTCTTCGAATGAAGAGAAAAATAGAGAA

ATCGCACGGCGGAGAGCCACCGATTCTCAGATGAATATCACAGGTCTCCACGATAGCACC

AGACTGAAT---------CAA------------------CAATCTTATTCACGC------

------TCCTATGAAAACCGGAATAGAAGATACAGTTCTGATGAAGACGATGATGAG---

------------------AATATGGCACCCCAGCGACGTCAA------------------

---CGCTCTCGGTCTCCT------------------------------------------

CCATCGTTTGCT------------------CACCACCAACGTCGAGATGATACCGGGTCG

TACTACAGAAGTCATCACACTCAAAATTCTTCAAATCAAAGAACTCATAACACTGATTTT

AGCTCGCATTATAGAGGGCAATACGGACCATCAACGTCGCAAAATGTGGGCATGCCATCA

AATGCTCAGAACGTGAGGATGCGCTCAGGAAAAAGCAGAGTCACAAAGACGCGTAGTCGC

AAGTGGCGACCTGGACAGAGAGCGCTTGAGGAAATTCGAAAATACCAAAAGTCCACCGAT

ATGCTGATTCAGAAAGCTCCCTTTGCACGTCTTGTCCACGAAATTATGCGCGAAGCAACT

TCGGAAAGTCAAGATTTTCGGATTCGTGCAGACGCTTTGATGGCTCTTCAAGAAGCGGCA

GAAGCGTTCATGGTGGAGATGTTCGAGGGATCCGTGTTGATTTGTAATCACGCGAAAAGA

GTAACTCTCATGCCGACAGATATTCAATTATATCGTCGCTTATGTCTTCGGAATCTTTCA

>C.species9

ATGTATCATCATGAC------------AGCGGCCCGCACATTGAAGAAGTTTTTGATCCA

CCACCGTCTCAAGAAACGATGCTTCGGGAAATA------------------------GCA

TCCCATCCCGATGTAATTGCACTCTCCAAAAAAGTCCGAAAAATCACAAAAATGCCGGAC

TCTGCCTTCATTTCTAGTGCCGATCGCCTAGTAGAAATAATCGATGCGTTTAGCGAGCAA

ATCGAAAAGTGGAAAGAAGATGAAACATTGGACGACCCATGCCCATATCTGTCATTGAAA

ATCGAATTTTTCACAGAAAAGAGAAACCAA------------------------TACCAA

AGAAAAAAT------------------------AGTTCGGCAGTTGATCGATACTATGAT

GGCAAAGATTCCCGGGATTACAGCAGCAGAAGGCCGTTGGAAGAGAGCCGAAGACGAGAG

GAGCCTCGAGATAGAGGTCATGAAACGAATATTGACATAACGCATCGTGGCGATAGCACC

AGTCTGAATCATTATTCGCAGCGACATTATTCTCAACGACAATCACAAAGTTCT------

------CGATTCGAA---------AGAGATCGTGAATCCGAAGAGAAA---GATGAG---

------------------AATAGGCACCCGAGACAACAATAT------------------

---CGTTCTAGATCTCCCCAA---------------------------------------

CACACACACAGCTACAACCAATCCACAATGCAT------CAGCGTGATGATACCAACGTT

TACCATAGAAGTCATCAA------------------------------------------

------------------------------AGCACGTCTCAG------------CCTTCA

------CAA---GTGAGAATGCGTTCTGGAAAAAGCCGTGTCACGAAGACACACAACCGC

AAGTTTCGACCCGGACAGAAAGCCTTAGCTGAAATTCGAAAGTATCAGAAGTCGACAGAT

ATGCTGATCCAGAAGGCTCCTTTTGCTCGTCTTGTTCATGAAATTGTTCGAGAACAAACC

AACCAAAGTAAAGACTATCGTATTCGTGCCGATGCTTTGATGGCTCTACAGGAAGCAGCA

GAAGCATTCATGGTTGAAATGTTCGAAGGATCCGTTCTGATTTGCAATCACGCTAAGCGT

GTCACACTCATGCCCACTGACATTCAGCTGTATCGTCGCTTGTGCCTCCGAAACCTATCC

>C.briggsae

ATGTACCATCATGAC------------AGCGGCCCGCACATTGAAGAAGTTTTTGATCCA

CCG---TCTCGACGAACGATGATGCAGGAAATA------------------------GAA

ACTCATCCCGATGTAATTGCATTCGGCAAAAAACTCCGAAAAATTAAAAACCAACCGGAG

TCCACCTTTCTTTCTAGTGCCGATCGCATGGAAGAAATAATCGATGCGTTTAGAGATCAA

ATCGCCAAGTGGGAAGAAGAGGAAGAGTTGAATGAGCCATGTGAATATCGGCAATTAAAA

ATTGAAATTTTCACGCAAAAGAAAATTGAA------------------------TACCAA

AGAAAAAAT------------------------AATTTGGCAGTCGACGAATTCTATAAG

AAGAGAAATCTTAAGAATCACAGCAACAGAAAACCGTTGGAAGAGAGCAGGAGAAGAGAG

GAGCCTCGAGATAGAGTCCACGAATCGAATATAGACATAACGCATCGTGGCGATAGCACC

AGTCTGAATCATTATTCTCGGCACCATTATTCTCAACGACAATCACAAAGTTCT------

------CGGTTCGAA---------AGAGAGCGTGAATCCGACGAGGAA---GAGGAA---

------------------AATAGTCAGCCGATTCAACGTTAT------------------

---CGTTCTAGATCTCCCAAA---------------------------------------

CCATCATACAGCTACAACCAATCGACAATGCAACAGTCACAACGTGATGATACCAACGTT

TACCATAGAAGCCATCAA------------------------------------------

------------------------------AGCACATCTCAA------------CCTCCA

------CAA---GTGAGAATGCGTTCCGGAAAAAGCCGTGTCACGAAGACACACAACCGC

AAGTTTCGACCTGGACAGAAAGCCTTGGCTGAAATTCGAAAGTATCAGAAGTCGACAGAT

ATGTTGATCCAGAAGGCTCCTTTTGTTCGTCTTGTTCATGAAATTATTCGAGAACAAACC

TACAAAAGTCAAGACTATCGTATTCGTGCGGATGCTTTGATGGCTCTACAGGAAGCAGCA

GAAGCATTCATGGTTGAAATGTTCGAAGGATCCGTACTGATTTGCAATCACGCTAAGCGT

GTCACACTCATGCCCACTGACATTCAGCTGTATCGTCGCTTGTGCCTTCGAAACCTATCC

>C.brenneri

ATGTTTCATCTCTCG------------GATGGTCCCACCATCGAAGAATTAGTGGACACA

CAACAATTAGAAAACACAGCAGAAGCTGAATTC------------------------AAA

GAAGAGCTTGATGTCATTAAG------AAAGAACTGGCAGCTGTCCTTGCCATTCCGGAT

------ATCCACAGAAACCGTGAAGCATTGGAAAAAAGTATCCGCATTCTAGAGAAAGCT

ATCGATAAATGGGAAGAGGATGAAGAAAACCAAGTATCACTCGAGCTTCGCAGGCAGTCT

ATTGGGAAGTTTAAAGAACAGCGTCGTAGC------------------------TGTAAA

CAGAAACTC------------------------CGAGACGCTGAAAATGCGTTTCACGAA

AGGAGAGAA---------------------CGGGAATATGAAGAAAGGACAATGAGAGAG

ATTCCTCGGAGGTACTCCAGTTTTCGGGATACAGACATAACGAGGCGTAACAATACGACA

GGACTATACCATCATTCTCAG------------------CAAAGTTCTTCTAAT------

------TTTCGGATG---------CAAGAATACAGTTCAGACGAAGAA---ATAGAA---

------------------AACATTCCAAGCTCACACCGCGAT------------------

---CGTTATAGGTTAGAAAAGTGTTTGATTATCGTTTTTCAAAATCTATATTTCAGTTAC

CCGCCAAAAAAAATCAGTCATTCAACAATGCTCCAACAACGCCGTGACATTAGCCCGGTT

GTTTATCGTAGTCAGCAA------------------------------------------

------------------------------CAGAGTTCAGCA------------GGTTCT

------CAACAAGAGCGGATGCGATCCGGAAAAAGTCGAGTAACAAAGACA---ACCCGC

AAGCACAGACCGGGGCAAAAAGCGTTGGCAGAGATAAGGAAATACCAGAAGTCAACTGAT

CTTTTGATTCAGAAAGCTCCATTTGCACGCCTTGTCCATGAAATTATCCGGGAAGCAACT

ACAAATAGTGGAGATTATCGCGTTCGTGCAGATGCTCTTCTAGCTCTCCAAGAAGGCGCT

GAAGCATTTATGGTTGAAATGTTTGAAGGATCTGTATTAATTTGTAACCACGCGAAGCGC

GTAACTCTTATGCCCACAGATATTCAATTATATCGACGTCTGTGCCTCAGAAATCTC---

>C.japonica

ATGCAACGA------ATGATAGAGATGGGCGGGCCTCACATAGAGGAAATTGTCGATCCG

CCGAGCCCGAGCAATTCTGTGCTTCAAGAAGCCGATTATCGACAAAATGGTCCGTCTCGC

AGTCGGCCAAAACTTATC---------GATCAAATTAGAACTCTTATTCGCACGCCAAAT

------TTCAACAAAGACGCCGTCAAGATGGGTCAAGCTATCGATCTAATGGAGCTGCAA

ATTGCCGAATGGGTTGAGGAGCAAATTCGATACGGATTCACACAGGAACGCGAAGATGCC

ATCTACCAGTACAGACGCAAATTGCGACGTCAAAACGCGAAACCTCGCCCACTTTTTTCA

CGCAAAAAATTCCAAATGTTCAAAAAAAGCGTCGAAGAAGCAGAAGAACGATACTTTGAA

GAAAGA---------------------------------GAGGAGAGCAGACGGCGAGAA

GAAGCACGCAGAGCAATGAGCTACAGTCGCGGGGACATTTCGGCACGAGACAATCGTTCA

AAACTACAT---------CAA------------------TCTCACACGCAAAGA------

------AATTACGGC---------GATAGCTTGGATTCGGACGATGAG---AATGAGCGT

GAGAATGGTTATCAGAGTTATAGACCGCCTCCACAGAGGCAACAACGTTTGCGTTCACGT

TCTCGATCCCGCTCTCCG------------------------------------------

ATGCGGTCGTCGTACCGTCATGAATCGCCCGAAAACAGCCGTAGAAACGCA---------

---------TCACATCAA------------------------------------------

------------------------------CAAACCGCACAA------------------

------------GTCAGAATGCGTGCCGGAAAGAACAACGTCACGAAAACA------AAA

AAATGGCGTCCAGGACAGAAGGCGTTGAGTGAGATTCGAAAATACCAAAATTCCACTGAT

TTGCTCATTCAAAAAGCCCCCTTCCGTCGATTAGTTCACCAGATTATTCAAGAAGCGACC

GGCTTCGATTCCGGATTCCGCATTCGCGCCGACGCGATGTCTGCCCTACAAGAAGCCGCC

GAGGCGTTCATCGTCGAGATGTTCGAGGGATCTGTTCTCATCTCGAATCACGCAAAACGG

GTCACTCTGATGACGGCCGACATTCAATTGTACCGTCGACTTTGCCTCCGAAATCTC---

>PS_C.elegans

------------------------------ACCCCAATTATTGAGGAAATCGCCGAG---

------------------------------------------------------------

---------------------------------ATGCAAAGAGTCACTTCAGTGCCGGGA

------TTCAACACAAGCGCCGCAGGTGTCAACGATTTGATCGACATTCTG---------

------------------------------------------------CAC---------

------------------------------------------------------------

---------------------------------AAGCAAGCCGAAGACGAATATCACGCG

CGAAAA------------------------------------------------------

------------------------TCGTCTATGGATTTCACGGTCGGCAGAAATTCCACG

AATCTT------------------------------------------------------

------------------------------------------------------------

------------------------------------------------------------

------------------------------------------------------------

------------------------------------------------------------

------------------------------------------------------------

------------------------------------------------------------

------------GTGCGGATGAGAGCCGGAAGGAACAGAGTCACCAAAACG------AGA

CGTTATAGACCGGGCCAGAAGGCATTGGAAGAGATCCGCAAGTACCAAAAAACTGAAGAC

CTTCTGATTCAAAAGGCTCCGTTCGCACGCCTCGTCCGCGAAATTATGCAGACTTCCACT

CCATTTGGCGCCGACTGCCGTATTCGTTCTGACGCCATCAGTGCTCTTCAAGAAGCGGCG

GAAGCATTTTTGGTCGAAATGTTCGAAGGATCGTCTCTTATATCCACCCATGCGAAACGT

GTCACACTCATGACAACGGATATTCAGTTATACAGACGTCTCTGCCTTCGACATCTC---

>PS_C.remanei

------------------------------GGACCGCGTATTGAAGAAATGGTGGAT---

------------------------------------------------------------

---------------------------------TATCGCAGAATCAGCCATCTTCCAGAC

------TTCAACAGAGACCCAGAATTAATCCAGGAGGTTATGAATTTGACA---------

------------------------------------------------CGT---------

------------------------------------------------------------

---------------------------------GAAACCGCAGAAGATGAATACTATACA

CGACGA------------------------------------------------------

------------------------TCTCAGATGAATATCACAGGTCTCCACGATAGCACC

AGACTG------------------------------------------------------

------------------------------------------------------------

------------------------------------------------------------

------------------------------------------------------------

------------------------------------------------------------

------------------------------------------------------------

------------------------------------------------------------

------------GTGAGGATGCGCTCAGGAAAAAGCAGAGTCACAAAGACG------CGC

AAGTGGCGACCTGGACAGAGAGCGCTTGAGGAAATTCGAAAATACCAAAAGTCCACCGAT

ATGCTGATTCAGAAAGCTCCCTTTGCACGTCTTGTCCACGAAATTATGCGCGAAGCAACT

TCGGAAAGTCAAGATTTTCGGATTCGTGCAGACGCTTTGATGGCTCTTCAAGAAGCGGCA

GAAGCGTTCATGGTGGAGATGTTCGAGGGATCCGTGTTGATTTGTAATCACGCGAAAAGA

GTAACTCTCATGCCGACAGATATTCAATTATATCGTCGCTTATGTCTTCGGAATCTT---

>PS_C.species9

------------------------------GGCCCGCACATTGAAGAAGTTTTTGAT---

------------------------------------------------------------

---------------------------------GTCCGAAAAATCACAAAAATGCCGGAC

------TTCATTTCTAGTGCCGATCGCCTAGTAGAAATAATCGATGCGTTT---------

------------------------------------------------CTG---------

------------------------------------------------------------

---------------------------------AGTTCGGCAGTTGATCGATACTATGAT

GGCAAA------------------------------------------------------

------------------------ACGAATATTGACATAACGCATCGTGGCGATAGCACC

AGTCTG------------------------------------------------------

------------------------------------------------------------

------------------------------------------------------------

------------------------------------------------------------

------------------------------------------------------------

------------------------------------------------------------

------------------------------------------------------------

------------GTGAGAATGCGTTCTGGAAAAAGCCGTGTCACGAAGACA------CGC

AAGTTTCGACCCGGACAGAAAGCCTTAGCTGAAATTCGAAAGTATCAGAAGTCGACAGAT

ATGCTGATCCAGAAGGCTCCTTTTGCTCGTCTTGTTCATGAAATTGTTCGAGAACAAACC

AACCAAAGTAAAGACTATCGTATTCGTGCCGATGCTTTGATGGCTCTACAGGAAGCAGCA

GAAGCATTCATGGTTGAAATGTTCGAAGGATCCGTTCTGATTTGCAATCACGCTAAGCGT

GTCACACTCATGCCCACTGACATTCAGCTGTATCGTCGCTTGTGCCTCCGAAACCTA---

>PS_C.briggsae

------------------------------GGCCCGCACATTGAAGAAGTTTTTGAT---

------------------------------------------------------------

---------------------------------CTCCGAAAAATTAAAAACCAACCGGAG

------TTTCTTTCTAGTGCCGATCGCATGGAAGAAATAATCGATGCGTTT---------

------------------------------------------------CGG---------

------------------------------------------------------------

---------------------------------AATTTGGCAGTCGACGAATTCTATAAG

AAGAGA------------------------------------------------------

------------------------TCGAATATAGACATAACGCATCGTGGCGATAGCACC

AGTCTG------------------------------------------------------

------------------------------------------------------------

------------------------------------------------------------

------------------------------------------------------------

------------------------------------------------------------

------------------------------------------------------------

------------------------------------------------------------

------------GTGAGAATGCGTTCCGGAAAAAGCCGTGTCACGAAGACA------CGC

AAGTTTCGACCTGGACAGAAAGCCTTGGCTGAAATTCGAAAGTATCAGAAGTCGACAGAT

ATGTTGATCCAGAAGGCTCCTTTTGTTCGTCTTGTTCATGAAATTATTCGAGAACAAACC

TACAAAAGTCAAGACTATCGTATTCGTGCGGATGCTTTGATGGCTCTACAGGAAGCAGCA

GAAGCATTCATGGTTGAAATGTTCGAAGGATCCGTACTGATTTGCAATCACGCTAAGCGT

GTCACACTCATGCCCACTGACATTCAGCTGTATCGTCGCTTGTGCCTTCGAAACCTA---

>PS_C.brenneri

------------------------------GGTCCCACCATCGAAGAATTAGTGGAC---

------------------------------------------------------------

---------------------------------CTGGCAGCTGTCCTTGCCATTCCGGAT

------ATCCACAGAAACCGTGAAGCATTGGAAAAAAGTATCCGCATTCTA---------

------------------------------------------------CGC---------

------------------------------------------------------------

---------------------------------CGAGACGCTGAAAATGCGTTTCACGAA

AGGAGA------------------------------------------------------

------------------------CGGGATACAGACATAACGAGGCGTAACAATACGACA

GGACTA------------------------------------------------------

------------------------------------------------------------

------------------------------------------------------------

------------------------------------------------------------

------------------------------------------------------------

------------------------------------------------------------

------------------------------------------------------------

------------GAGCGGATGCGATCCGGAAAAAGTCGAGTAACAAAGACA------CGC

AAGCACAGACCGGGGCAAAAAGCGTTGGCAGAGATAAGGAAATACCAGAAGTCAACTGAT

CTTTTGATTCAGAAAGCTCCATTTGCACGCCTTGTCCATGAAATTATCCGGGAAGCAACT

ACAAATAGTGGAGATTATCGCGTTCGTGCAGATGCTCTTCTAGCTCTCCAAGAAGGCGCT

GAAGCATTTATGGTTGAAATGTTTGAAGGATCTGTATTAATTTGTAACCACGCGAAGCGC

GTAACTCTTATGCCCACAGATATTCAATTATATCGACGTCTGTGCCTCAGAAATCTC---

>PS_C.japonica

------------------------------GGGCCTCACATAGAGGAAATTGTCGAT---

------------------------------------------------------------

---------------------------------ATTAGAACTCTTATTCGCACGCCAAAT

------TTCAACAAAGACGCCGTCAAGATGGGTCAAGCTATCGATCTAATG---------

------------------------------------------------CGC---------

------------------------------------------------------------

---------------------------------GAAGAAGCAGAAGAACGATACTTTGAA

GAAAGA------------------------------------------------------

------------------------AGTCGCGGGGACATTTCGGCACGAGACAATCGTTCA

AAACTA------------------------------------------------------

------------------------------------------------------------

------------------------------------------------------------

------------------------------------------------------------

------------------------------------------------------------

------------------------------------------------------------

------------------------------------------------------------

------------GTCAGAATGCGTGCCGGAAAGAACAACGTCACGAAAACA------AAA

AAATGGCGTCCAGGACAGAAGGCGTTGAGTGAGATTCGAAAATACCAAAATTCCACTGAT

TTGCTCATTCAAAAAGCCCCCTTCCGTCGATTAGTTCACCAGATTATTCAAGAAGCGACC

GGCTTCGATTCCGGATTCCGCATTCGCGCCGACGCGATGTCTGCCCTACAAGAAGCCGCC

GAGGCGTTCATCGTCGAGATGTTCGAGGGATCTGTTCTCATCTCGAATCACGCAAAACGG

GTCACTCTGATGACGGCCGACATTCAATTGTACCGTCGACTTTGCCTCCGAAATCTC---
